# Discovery of electrochemically induced grain boundary transitions

**DOI:** 10.1038/s41467-021-22669-0

**Published:** 2021-04-22

**Authors:** Jiuyuan Nie, Chongze Hu, Qizhang Yan, Jian Luo

**Affiliations:** 1 Department of Nanoengineering, University of California, San Diego, La Jolla, CA USA; 2 Program of Materials Science and Engineering, University of California, San Diego, La Jolla, CA USA

**Keywords:** Energy science and technology, Materials science, Surfaces, interfaces and thin films, Theory and computation, Phase transitions and critical phenomena

## Abstract

Electric fields and currents, which are used in innovative materials processing and electrochemical energy conversion, can often alter microstructures in unexpected ways. However, little is known about the underlying mechanisms. Using ZnO-Bi_2_O_3_ as a model system, this study uncovers how an applied electric current can change the microstructural evolution through an electrochemically induced grain boundary transition. By combining aberration-corrected electron microscopy, photoluminescence spectroscopy, first-principles calculations, a generalizable thermodynamic model, and ab initio molecular dynamics, this study reveals that electrochemical reduction can cause a grain boundary disorder-to-order transition to markedly increase grain boundary diffusivities and mobilities. Consequently, abruptly enhanced or abnormal grain growth takes place. These findings advance our fundamental knowledge of grain boundary complexion (phase-like) transitions and electric field effects on microstructural stability and evolution, with broad scientific and technological impacts. A new method to tailor the grain boundary structures and properties, as well as the microstructures, electrochemically can also be envisioned.

## Abstract (web summary)

Electric fields and currents can alter microstructures of materials in unexpected ways. Here the authors report how electrochemical reduction can cause a grain boundary disorder-to-order transition and show the electric field effects on microstructural stability and evolution.

## Introduction

It has been long proposed^1^ that grain boundaries (GBs) can be considered as two-dimensional (2D) interfacial phases (a.k.a. “complexions”^2–7^), which can undergo transitions to influence various kinetic, mechanical, chemical, electronic, ionic, and other properties^3–5,7–12^. However, prior studies have seldom elucidated the mechanism of how an external stimulus—other than (a very few instances of) temperature or segregation—can induce a GB transition and subsequently alter the properties with clear underlying mechanisms. Notably, it was proposed that GB transitions can alter microstructural evolution abruptly^3,4,7^. Also, interestingly, electric fields and currents, which are used in various innovative materials processing^13–16^ and electrochemical energy conversion^17,18^ and storage^19^ devices, can often alter microstructures unexpectedly and abruptly. Yet, the underlying atomic-level mechanisms remain elusive.

This study first aims at decoding how an electric field/current can alter microstructural evolution, an outstanding scientific problem of fundamental interest yet with broad technological implications. A spectrum of fascinating and intriguing observations of the “electric field effects” of suppressed^20,21^ vs. enhanced^17,20–25^ (including abnormal^17,23^) grain growth has been made in several oxides. See Supplementary Note 1 for elaboration, along with a discussion of relevant materials processing technologies^13–16^ (including methods to sinter ceramics in seconds^13,16^) where electric fields/currents can affect microstructural evolution. Moreover, electric fields and currents are present in solid oxide fuel cells^17,18^, solid-state batteries^19^, and various other electrochemical and electronic devices, where they can cause unexpected (often undesirable or even catastrophic) changes in microstructures or GB properties.

In a broader context, the formation and transition of 2D interfacial phases^1^, which were also named as “complexions”^2–7^ to differentiate them from thin layers of precipitated 3D bulk phases at GBs, can often control various materials properties^3–5,7–12^. However, the majority of prior studies focused on symmetric tilt or twist GBs that are relatively easy to image and model. For example, a most recent study observed GB phase transitions at a symmetric tilt GB in pure copper^26^. General GBs (asymmetric GBs that are often of mixed tilt and twist characters^27^) are much less understood, but they are ubiquitous and can often be the weaker links mechanically and chemically in polycrystalline materials^9,11,12^. Moreover, Dillon and Harmer proposed that anisotropic complexion transitions at general GBs can cause the AGG^3,4,7^, which shed light on one of the most long-standing mysteries in materials science but open questions remain. Specifically, how an abnormal grain can initiate remains under debate for nearly a century, albeit the cause can vary for different cases.

In this study, we use ZnO-Bi_2_O_3_ as a model system to uncover how an applied electric current can change the microstructural evolution (including triggering AGG) through electrochemically induced transitions at general GBs. Here, we combine aberration-corrected electron microscopy, first-principles calculations, and ab initio molecular dynamics (AIMD) to reveal that electrochemical reduction can cause GB disorder-to-order transitions to markedly increase GB diffusivities. Consequently, enhanced or abnormal grain growth takes place. A generalizable thermodynamic model is further proposed. This work builds a bridge between two important areas of GB complexion (phase-like) transitions and electric field effects on microstructural stability and evolution, while significantly advancing our fundamental knowledge in both areas. Furthermore, the discovery opens a new window to tailor a broad range of GB (e.g., electronic or ionic) properties, as well as the microstructures, electrochemically.

## Results

### Characteristic amorphous-like GBs in ZnO-Bi_2_O_3_

**Fig. 1:**
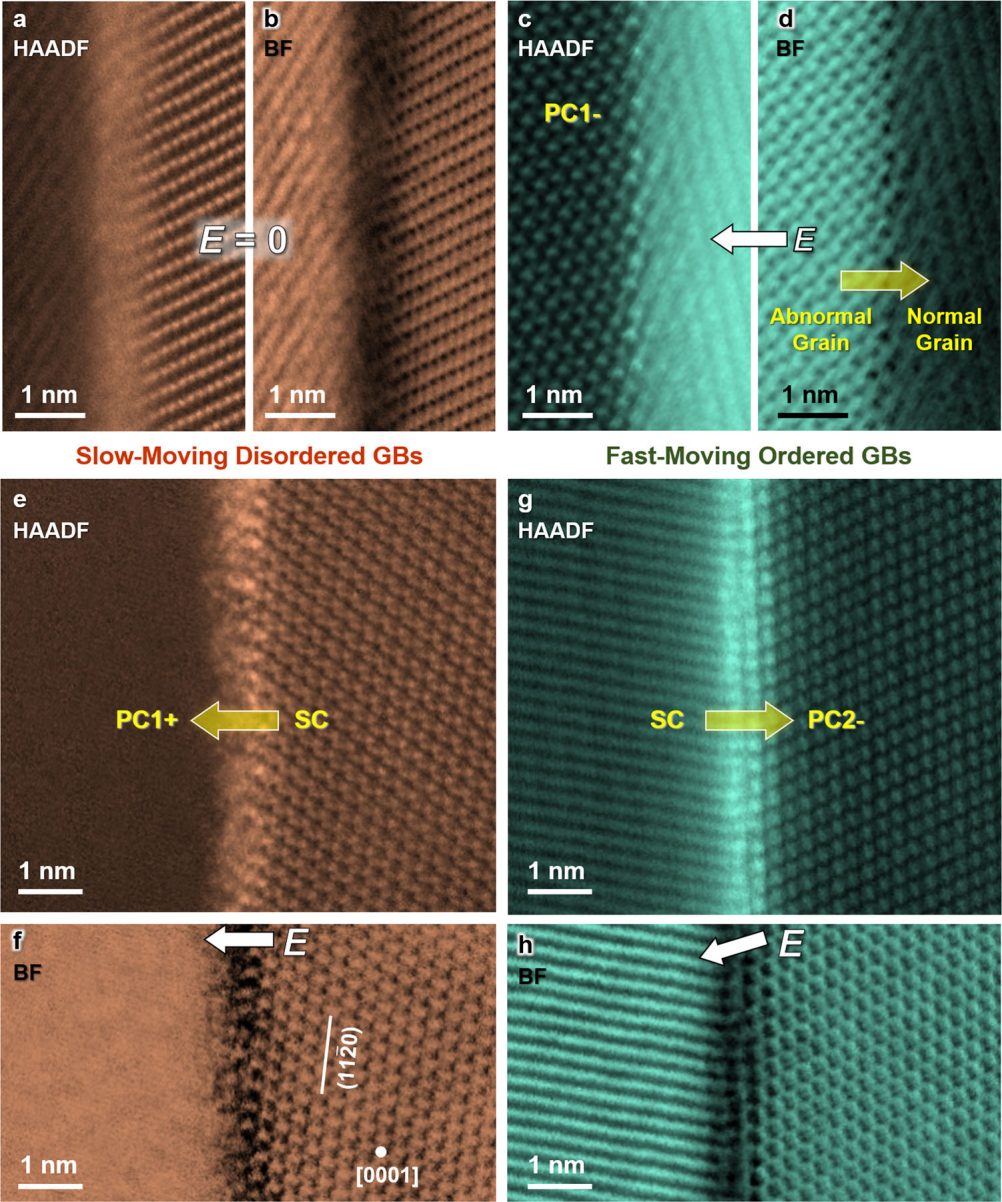
AC STEM high-angle annular dark-field (HAADF) and bright-field (BF) images of representative slow-moving disordered vs. fast-moving ordered GBs. **a**, **b** A nanoscale amorphous-like IGF (a.k.a. disordered GB) observed in a reference specimen annealed without an electric field, which is characteristic of all general GBs in Bi_2_O_3_-saturated ZnO^29^. STEM images of **c**, **d** an ordered GB of an abnormal grain in the electrochemically reduced PC1– region, as well as **e**, **f** a slow-moving disordered GB at the oxidized PC1+/SC interface vs. **g**, **h** a fast-moving ordered GB at the reduced SC/PC2– interface in a PC1/SC/PC2 sandwich specimen annealed with an applied electric current (as schematically shown in Fig. 2a). STEM images for additional examples can be found in Supplementary Figs. 9–12, showing the generality of the observations of slow-moving disordered GBs vs. fast-moving ordered GBs.

Here, we select Bi_2_O_3_-doped ZnO (with 0.5 mol% Bi_2_O_3_ added in the polycrystal) as our model system. The solid solubility limit of Bi_2_O_3_ in the ZnO crystal is <0.06 mol%^28^ so that most Bi_2_O_3_ is present at GBs and triple-grain junctions^29^. The formation of ~0.7–0.9 nm thick liquid-like intergranular films (IGFs) at general GBs is confirmed in our reference specimen without an applied electric field by aberration-corrected scanning transmission electron microscopy characterization (AC STEM) and high-resolution transmission electron microscopy (Fig. 1a, b). Such nanometer-thick IGFs, which have been equivalently interpreted as equilibrium-thickness liquid-like interfacial films by Clarke^30^ or disordered multilayer adsorbates by Cannon et al.^31^, represent one most widely observed complexion in ceramics^4,5,8,29–31^. These IGFs are also called “amorphous-like” or “disordered” GBs here, albeit the existence of partial orders^5,6^. Prior studies have demonstrated that such amorphous-like IGFs form at all general GBs in Bi_2_O_3_-saturated ZnO at thermodynamic equilibria both above and below the bulk eutectic temperature^29^. Surprisingly and interestingly, here we observe highly ordered GB structures in ZnO-Bi_2_O_3_ (Fig. 1c, d, g, h) that have never been reported before.

### Electrochemical reduction enhanced grain growth

**Fig. 2:**
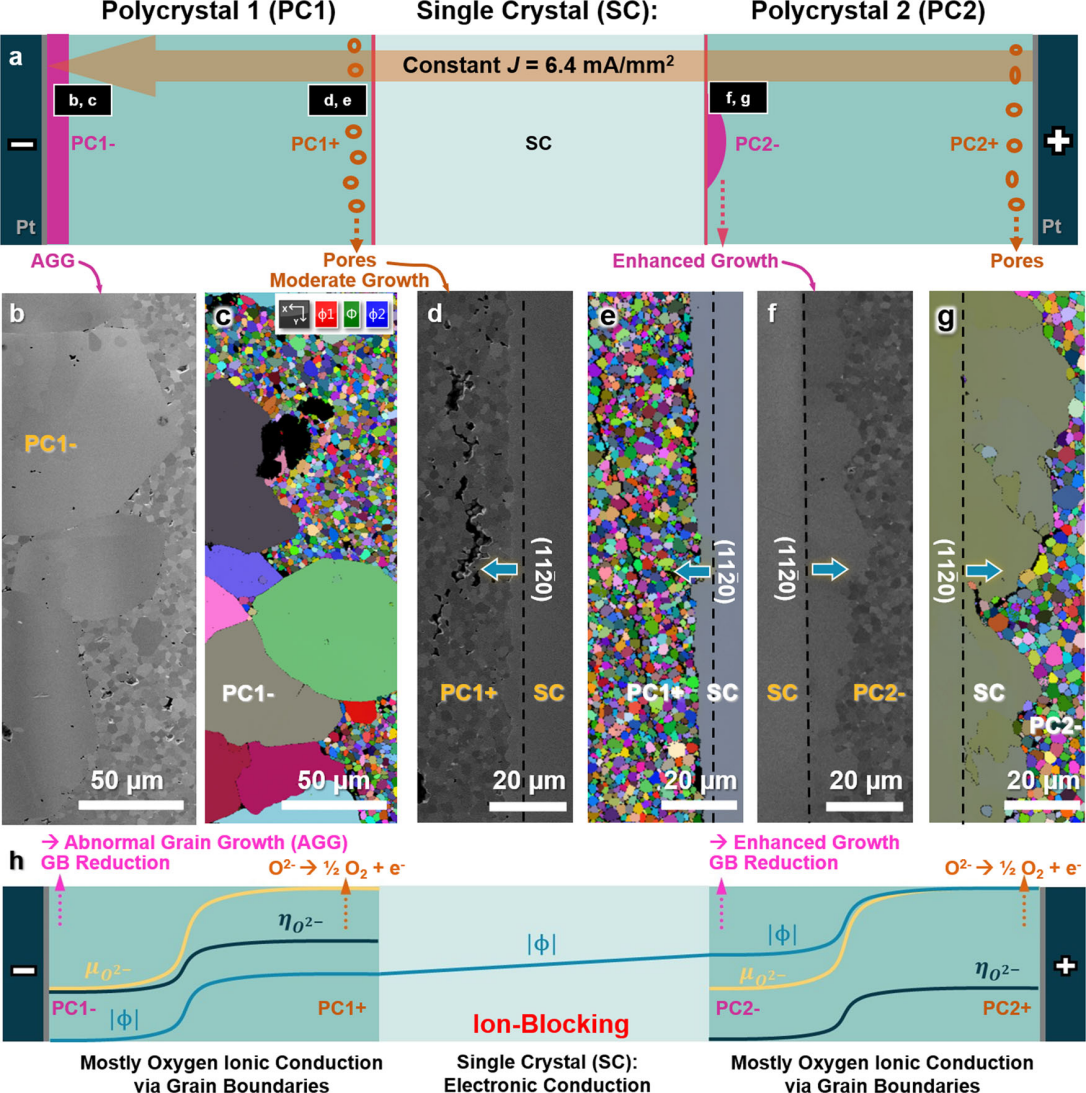
Microstructure of a Bi_2_O_3_-doped ZnO sandwich specimen isothermally annealed under a constant applied current. **a** A schematic illustration of this PC1/SC/PC2 sandwich specimen. Enhanced or abnormal grain growth takes place in the reduced PC1– and PC2– regions. Cross-sectional scanning electron microscopy (SEM) micrographs and electron backscatter diffraction (EBSD) Euler maps of **b**, **c** abnormal grain growth (AGG) near the cathode in the reduced PC1– region, as well as **d**, **e** moderate migration of the oxidized PC1+/SC interface vs. **f**, **g** enhanced migration of the reduced SC/PC2– interface. Dashed lines indicate the original positions of the SC/PC interfaces. See Supplementary Fig. 2 for quantitative measurements of migration distances of both PC1+/SC and SC/PC2– interfaces (in comparison with those in a reference sandwich specimen without an applied electric field) and Supplementary Figs. 3–5 for addition EBSD maps. **h** Schematic profiles of electric ($\phi$), chemical (${\mu }_{{O}^{2-}}$), and electrochemical (${\eta }_{{O}^{2-}}$) potentials vs. locations. See Supplementary Fig. 1 for further detail.

We design and fabricate a polycrystal 1/single crystal/polycrystal 2 (PC1/SC/PC2) sandwich specimen to conduct a grain growth experiment with a constant current density of 6.4 mA/mm^2^ (Fig. 2a and Supplementary Fig. 1 for more details). First, we observe AGG in the PC1– region (where “–” vs. “+” denotes the reduced vs. oxidized region) near the negative electrode (cathode), as evident in Fig. 2b, c. Second, we observe enhanced migration of the SC/PC2– interface (Fig. 2f, g) vs. only moderate migration of the PC1+/SC interface (Fig. 2d, e). Quantitative measurements of the migration distances (shown in Supplementary Fig. 2) reveal abruptly enhanced migration at the (most reduced) middle section of the SC/PC2– interface, in comparison with the (oxidized) PC1+/SC interface and both interfaces in a reference sandwich specimen annealed with no electric field. A detailed and quantitative comparison can be found in Supplementary Note 2.

**Fig. 3:**
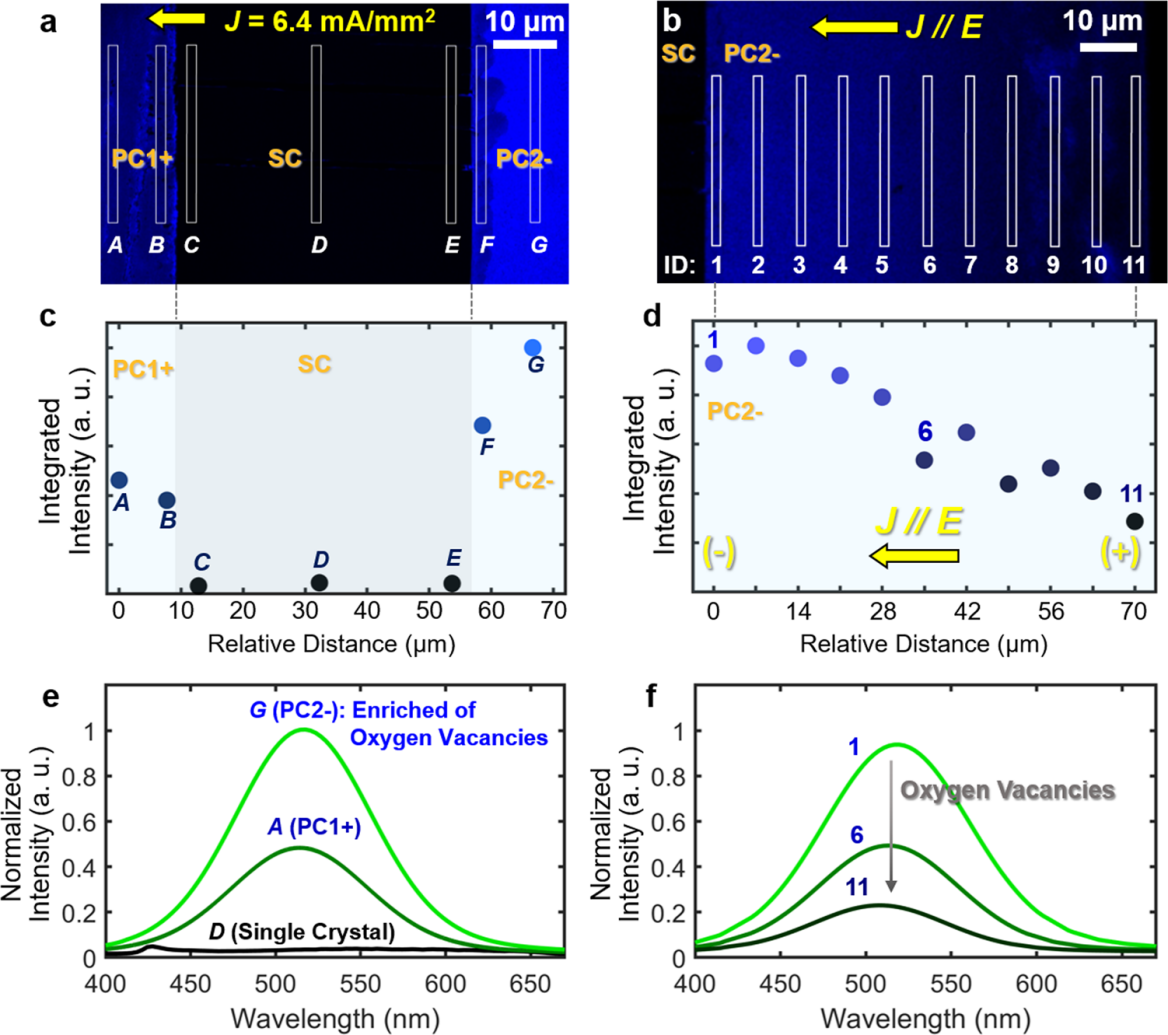
Photoluminescence spectroscopy of the sandwich specimen annealed with a constant applied electric current, suggesting the enrichment of oxygen vacancies in the reduced PC2– region. **a**, **b** Maps of the photoluminescence intensity at the 526 nm wavelength of the cross-sectional PC1/SC/PC2 sandwich specimen. **c**, **d** Integrated photoluminescence intensities for the combined photoluminescence peak at ~400–700 nm at different locations, representing all defects at GBs. Photoluminescence spectra are collected at Locations *A*–*G* labeled in **a** and Locations 1–11 labeled in **b**, respectively. **e**, **f** The normalized photoluminescence intensity vs. wavelength curves of decomposed green-emission band (representing the oxygen vacancy concentration) at selected locations. See Supplementary Note 4 and Supplementary Fig. 8 for further detail. In **c**–**f**, a.u. stands for arbitrary unit.

Next, let us show that the PC1– and PC2– regions in our sandwich specimen (Fig. 2), where we observe increased GB mobilities (either AGG or enhanced migration of the SC/PC interface), are oxygen reduced. Here, Bi_2_O_3_-enriched liquid-like IGFs in polycrystals are ion-conducting, while the ZnO single crystal is electron-conducting but ion-blocking (see Supplementary Note 3). Hence, PC1+ and PC2+ regions are oxidized (as the oxygen ions are blocked and accumulated near the interfaces), which is evident by the pore formation (see, e.g., Fig. 2d) due to an oxidation reaction that produces O_2_. On the other hand, the PC1– and PC2– regions must be reduced (due to the depletion of oxygen ions). Subsequently, we can sketch the profiles of electric ($\phi$), chemical (${\mu }_{{O}^{2-}}$), and electrochemical (${\eta }_{{O}^{2-}}$) potentials vs. locations in Fig. 2h according to a model presented in Supplementary Note 3. The electrochemical reduction in the PC1– region near the negative electrode is well expected. To prove the reduction in the PC2– region, we conduct spatially resolved photoluminescence spectroscopy to probe oxygen vacancies (Fig. 3a). The integrated photoluminescence intensities for the combined photoluminescence peak at ~400–700 nm (representing all defects at GBs) at different locations are shown in Fig. 3c, d. We further decompose this combined peak into a “green” band that is known to represent the oxygen vacancies and an “orange” band that most likely represents Zn interstitials (see Supplementary Note 4 and Supplementary Fig. 8). The decomposed green-band peaks at selected locations shown in Fig. 3e, f reveal the enrichment of oxygen vacancies to prove reduction in the PC2– region (that leads to enhanced migration of the SC/PC2– interface).

**Fig. 4:**
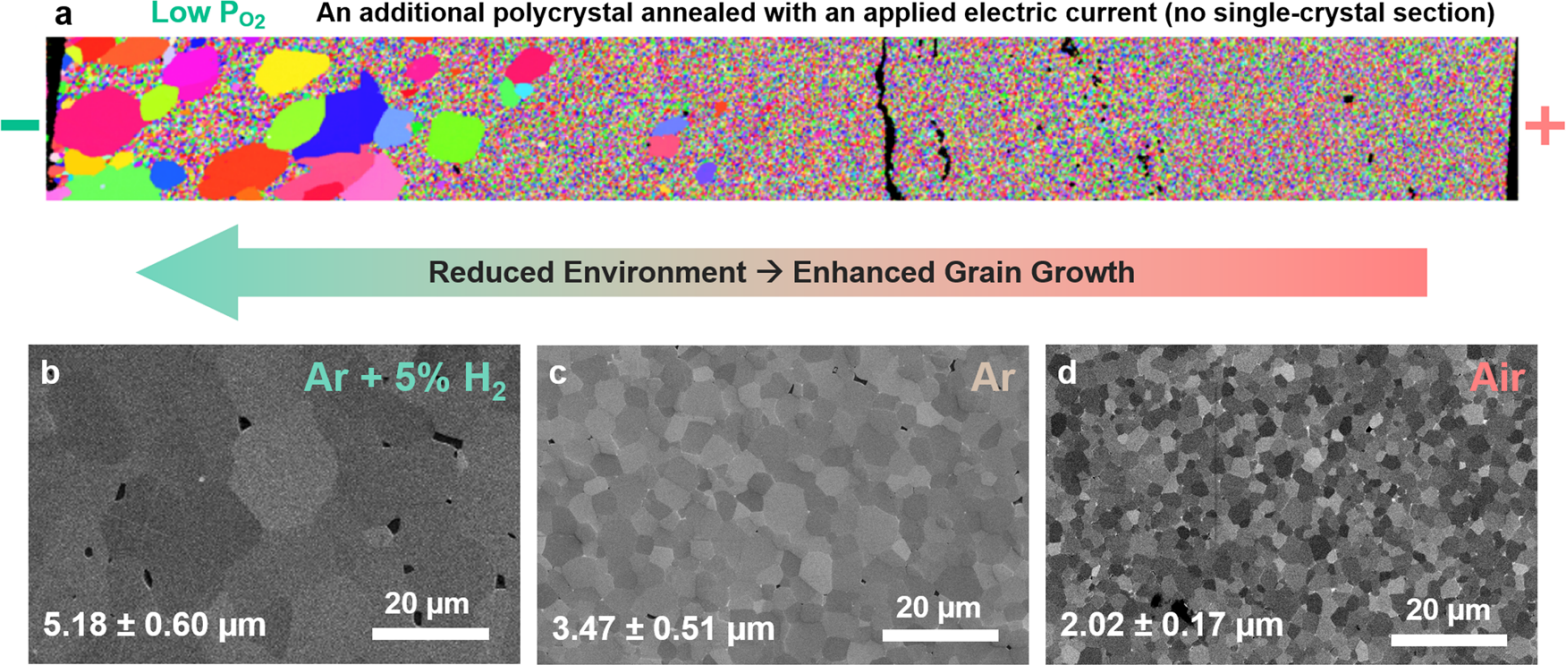
Enhanced grain growth in reduced environments for ZnO + 0.5 mol% Bi_2_O_3_ polycrystalline specimens. **a** EBSD map of a ZnO + 0.5 mol% Bi_2_O_3_ polycrystalline specimen (without a single-crystal section) annealed with an applied current, showing similar abnormal grain growth in the reduced region near the cathode (negative electrode). Cross-sectional SEM micrographs of polycrystalline specimens quenched from 880 °C after annealing for 4 h in **b** Ar + 5% H_2_ **c** Ar, and **d** air. Additional low-magnification images are shown in Supplementary Fig. 19. These observations further support that the oxygen reduction caused enhanced grain growth.

Moreover, we show that (i) an applied electric current can also induce AGG near the cathode (reduced region) in a simple polycrystalline specimen (Fig. 4a), and (ii) the grain growth of ZnO + 0.5 mol% Bi_2_O_3_ polycrystals can also be enhanced in reduced atmospheres (Ar-H_2_ and Ar vs. air) without an electric field (Fig. 4b–d). These observations further support that the oxygen reduction (instead of the electric field) is the direct reason that promotes grain growth.

### Reduction-induced GB disorder-to-order transitions

To probe the atomic-level mechanism of the reduction-induced increases of GB mobilities, we further examine the underlying GB structures by AC STEM (Fig. 1). On the one hand, the slow-moving oxidized PC1+/SC interface is disordered (Fig. 1e, f), similar to the characteristic amorphous-like IGFs observed in a reference specimen with no electric field (Fig. 1a, b) that also exhibits similar low mobility (see Supplementary Note 2). On the other hand, the fast-moving and reduced PC2–/SC interface shows highly ordered structures (Fig. 1g, h). Furthermore, the GBs of the fast-growing abnormal grains in the reduced PC1– region are also ordered (Fig. 1c, d).

Additional examples of these four cases are given in Supplementary Figs. 9–12 to show the generality of the observations, and they are further discussed in Supplementary Note 5. Notably, various monolayer-, bilayer-, and trilayer-like GB structures (presumably dependent on the specific crystallographic characters of the general GBs randomly selected from the specimen) have been observed in the reduced regions, but they are all ordered with high mobilities (in contrast to slow-moving amorphous-like GBs). Thus, we conclude that electrochemical reduction can generally induce disorder-to-order GB structural transitions with increased mobilities.

**Fig. 5:**
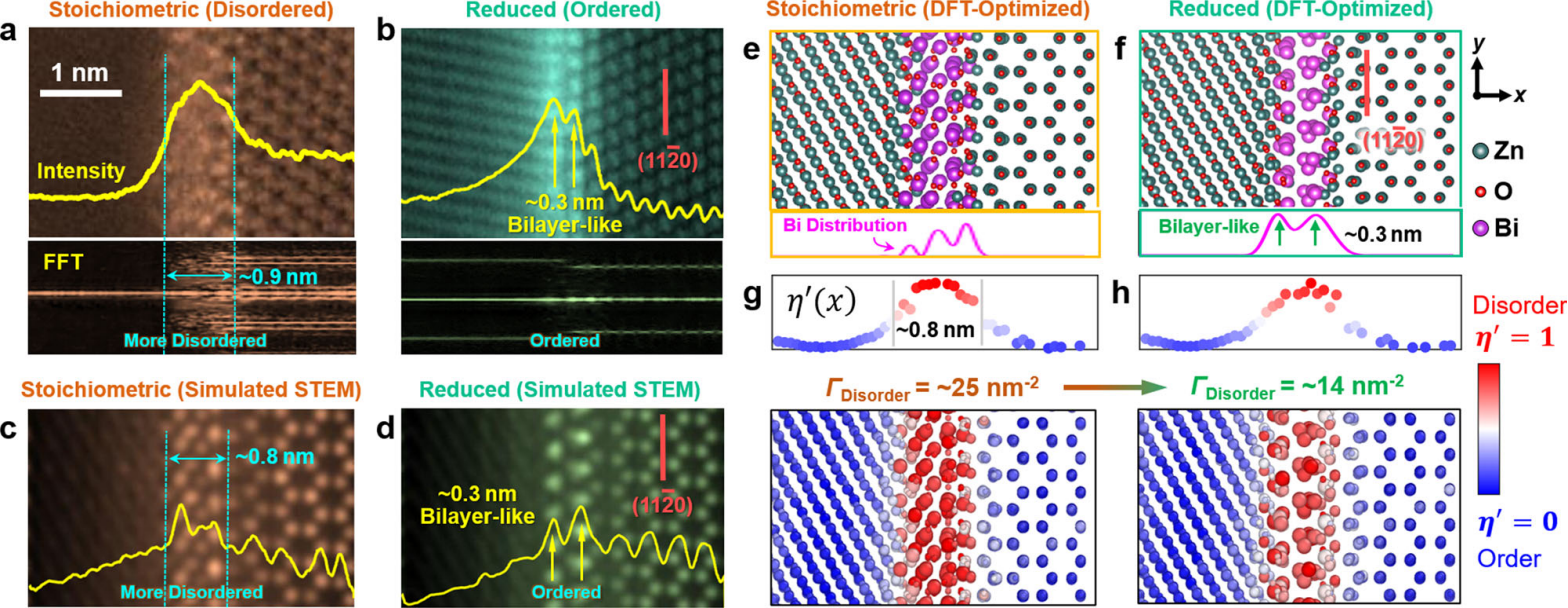
Comparison of experiments and DFT simulations of stoichiometric (disordered) vs. reduced (ordered) GB structures and AIMD-simulated GB diffusivities. Expanded experimental STEM HAADF images for **a** a disordered and stoichiometric GB vs. **b** an ordered (bilayer-like) and reduced GB; here, we plot the averaged HAADF intensity and line-by-line FFT patterns to illustrate the layering and periodic orders. Simulated STEM HAADF images for **c** stoichiometric GB vs. **d** reduced GB, respectively, based on DFT-optimized structures of **e** the stoichiometric vs. **f** reduced GBs, where the projected Bi density profiles are plotted beneath. The calculated disorder parameters for all atoms for DFT-optimized **g** stoichiometric vs. **h** reduced GB structures. The projected disorder parameter profiles ($\eta {\prime} (x)$) are shown above. The GB excess of disorder (computed by integrating $\eta {\prime} (x)$) decreases from *Γ*_Disorder_ = ~25 nm^−2^ for the stoichiometric GB to *Γ*_Disorder_ = ~14 nm^−2^ for the reduced GB, thereby showing that the oxygen reduction induces GB ordering.

Figure 5a, b further compares a pair of enlarged AC STEM high-angle annular dark-field (HAADF) images of GB structures at the oxidized PC1+/SC and the reduced SC/PC2– interfaces. Here, we also conduct image analyses to illustrate the layering and periodic orders (see Methods). While the amorphous-like GB (Fig. 5a) does show some partial orders (well-known for this type of IGFs^5,6^), it is more disordered and wider than the reduced GB that is highly ordered and bilayer-like in the case shown in Fig. 5b.

### Validation by first-principles calculations

Density functional theory (DFT) calculations are critically compared with AC STEM images to further verify the reduction-induced GB disorder-to-order transition. Note that the bright contrasts at ZnO GBs in the HAADF images (see Fig. 1) are due to heavy Bi adsorbates (because of the *Z* contrast). Such a strong Bi segregation has also been directly verified by energy dispersive X-ray spectroscopy (EDS), as shown in Supplementary Fig. 13. Based on the STEM images, we first construct an asymmetric GB to represent a general GB for DFT calculations, where one terminal plane was set to $(11\bar{2}0)$ to mimic both SC/PC interfaces observed in the STEM images (Fig. 5a, b). Next, the Bi coverage with a value of *Γ*_Bi_ = ~11.7 nm^−2^ was adopted to match prior experimental measurements of Bi segregation at ZnO GB at ~840–880 °C^28^ (see more details in Methods and Supplementary Note 6).

DFT structural optimization shows that the stoichiometric GB (representing oxidized conditions in experiments) exhibits a more disordered structure (Fig. 5e), while a reduced GB (after removing approximately one monolayer of oxygen) exhibits a more ordered bilayer-like structure (Fig. 5f; matching the STEM image in Fig. 5b). The bilayer-like Bi adsorption can be evident in the projected Bi distribution profile shown below Fig. 5f, with the interlayer distance of ~0.3 nm matching the STEM measurement (Fig. 5b). The simulated STEM images based on DFT-optimized structures (Fig. 5c, d) further verified disordered interfacial structure in stoichiometric GBs but ordered and bilayer-like Bi segregation structure in reduced GBs. Moreover, we calculate a structural disorder parameter (${{\eta }}^{{\prime} }$) for each atom in the DFT-relaxed structures and plot the projected disorder profiles (${{\eta }}^{{\prime} }(x)$) in Fig. 5g, h. For the disordered GB, the interfacial width is calculated to be ~0.8 nm based on the ${{\eta }}^{{\prime} }(x)$ profile (Fig. 5g) and intensity profile in simulated STEM image (Fig. 5c), which agrees with STEM measured value of ~0.9 nm as shown in Fig. 5a (and ~0.7–0.9 nm for the different disordered GBs observed in this study). Further quantifications (by integrating the ${{\eta }}^{{\prime} }(x)$ profiles) show that the stoichiometric GB has larger GB excess of disorder (*Γ*_Disorder_ ≈ 25 nm^−2^) than the reduced GB (~14 nm^−2^); in other words, oxygen reduction makes the GB structure more ordered, which agrees with the experiments (Fig. 5a vs. 5b). We further simulated STEM images from DFT-optimized GBs (Fig. 5c, d), which are consistent with the experiments (Fig. 5a, b) for the general disordered (Fig. 5a, c) vs. ordered (Fig. 5b, d) characters.

DFT calculations have also been conducted for GBs of different levels of Bi adsorption and oxygen reduction; see Supplementary Note 6 and Supplementary Figs. 14 and 15 for further details.

**Fig. 6:**
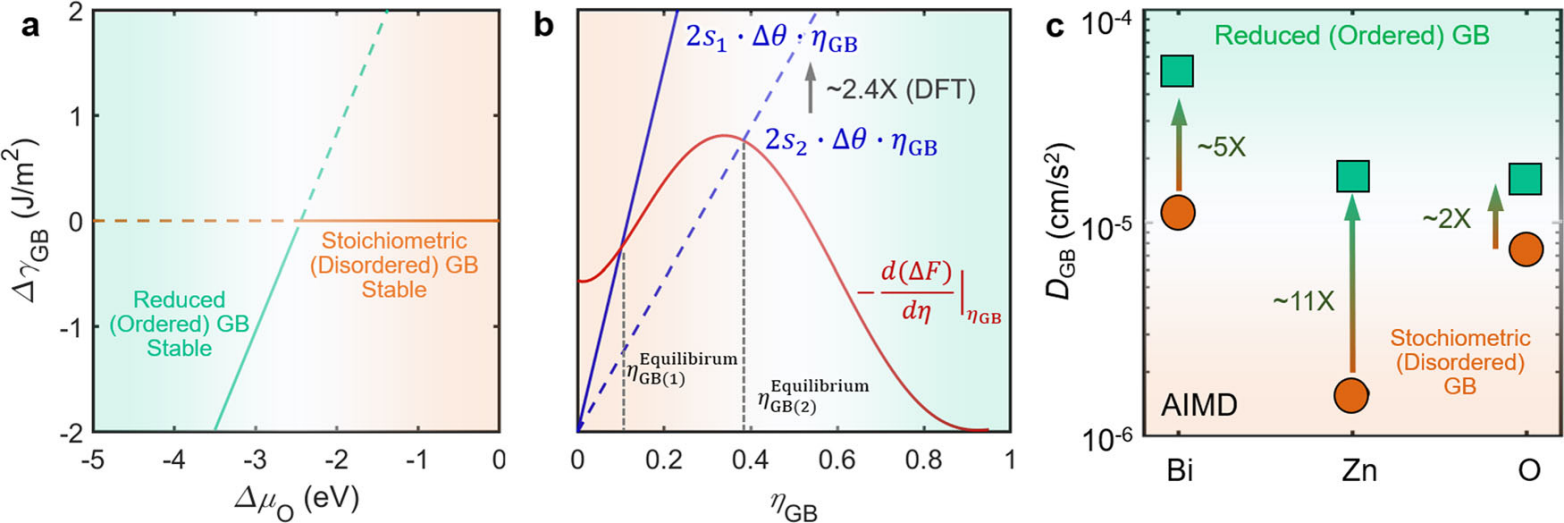
DFT-calculated interfacial energetical diagram, schematic diagram of GB disorder-to-order transition based on a generalized thermodynamic model, and ab initio molecular dynamics (AIMD)-simulated GB diffusivities. **a** Computed GB energy difference ${\triangle \gamma }_{{\rm{GB}}}$ (≡ ${\gamma }_{{\rm{GB}}}^{{\rm{Reduced}}}-{\gamma }_{{\rm{GB}}}^{{\rm{Stoichiometric}}}$) vs. oxygen chemical potential difference $\triangle {\mu }_{{\rm{O}}}$ (${\rm{\equiv }}{\mu }_{{\rm{O}}}-\frac{1}{2}{E}_{{{\rm{O}}}_{2}}$), showing a transition from the stoichiometric (disordered) GB to the reduced (ordered) GB with decreasing oxygen chemical potential, consistent with experiments. **b** Schematic illustration of a graphical construction method to solve Eq. (2). The intersection of the red and blue lines indicates an equilibrium order parameter, e.g., ${\eta }_{{\rm{GB}}(1)}^{{\rm{Equilibrium}}}$. DFT calculations showed that the parameter *s* for the stoichiometric GB (*s*_1_) is about ~2.4× of reduced GB (*s*_2_), suggesting that oxygen reduction can lead to larger equilibrium order parameter ${\eta }_{{\rm{GB}}(2)}^{{\rm{Equilibrium}}}$ and thus explain the GB disorder-to-order transition. **c** GB diffusivities calculated by AIMD simulations. The GB diffusivities in the reduced (ordered) GB are markedly increased in comparison with those in the stoichiometric (disordered) GB.

Furthermore, we calculate GB energies using the procedure described in Supplementary Note 7. Figure 6a shows the DFT-computed GB energy difference ${\triangle \gamma }_{{\rm{GB}}}$ (≡ ${\gamma }_{{\rm{GB}}}^{{\rm{Reduced}}}-{\gamma }_{{\rm{GB}}}^{{\rm{Stoichiometric}}}$) as a function of oxygen chemical potential difference $\triangle {\mu }_{{\rm{O}}}$ for both stoichiometric and reduced GBs. The crossover of two ${\triangle \gamma }_{{\rm{GB}}}$ curves implies a GB phase-like or complexion transition from the disordered and stoichiometric GB (as shown by the orange solid line in Fig. 6a) to the ordered and reduced GB (as shown by the green solid line in Fig. 6a) with decreasing oxygen chemical potential, consistent with our experimental observations.

### A generalizable thermodynamic model

To further discuss the physical origin of the reduction-induced GB disorder-to-order transition, we adopt a generalizable thermodynamic model following Tang et al.^2^. Here, the interfacial excess grand potential for a two-component GB in a diffuse-interface model is given by^2^:

1 $${\sigma }^{x}= \int_{-\infty }^{+\infty }\left[\varDelta f(\eta ,c)+\frac{{\kappa }_{\eta }^{2}}{2}\cdot {\left(\frac{d\eta }{dx}\right)}^{2}+\frac{{\kappa }_{c}^{2}}{2}\cdot {\left(\frac{dc}{dx}\right)}^{2}+s\cdot g(\eta )\cdot \left|\frac{d\theta }{dx}\,\right|\right]dx,$$

where concentration *c*(*x*), crystallinity *η*(*x*) ($=1-{\eta }^{\prime} (x)$), and crystallographic orientation *θ*(*x*) are functions of the spatial variable *x*, *Δf*(*c, η*) is the homogenous-free energy density referenced to the equilibrium bulk phases, and *κ*_*η*_, *κ*_*c*_, and *s* are gradient energy coefficients. As derived in Supplementary Note 8, minimization of Eq. (1) leads to:

2 $${\sigma }^{x}=s\cdot \varDelta \theta \cdot {\eta }_{{\rm{GB}}}^{2}+\varDelta F({\eta }_{{\rm{GB}}}),$$

where *η*_GB_ is the order parameter at the center of the GB located at *x* = 0. Here, the first term *s*·Δ*θ*·*η*^2^_GB_ represents an energetic penalty to have a GB misorientation Δ*θ*, which can be lowered by GB disordering. The second term represents the total increased free energy due to the formation of a diffuse interface, which (initially) increases with GB disordering. Thus, the equilibrium level of GB disorder, ${\eta^{\prime}} _{{\rm{GB}}}^{{\rm{Equilibrium}}}{\rm{\equiv }}1-{\eta }_{{\rm{GB}}}^{{\rm{Equilibrium}}}$ is determined by a tradeoff between these two terms. Specifically, Eq. (2) suggests that a smaller *s*·Δ*θ* will result in a more ordered GB at equilibrium state ($d\left({\sigma }^{x}\right)/d({\eta }_{{\rm{GB}}}^{{\rm{Equilibrium}}})=0$). This is further illustrated in Fig. 6b using a graphical construction method to solve Eq. (2) following Cahn’s critical point wetting model^32^, as described in Supplementary Note 8.

Subsequently, we have developed a DFT-based method to estimate *s*·Δ*θ* to show that it (or *s* since Δ*θ* is a constant) can be decreased by about 2.4 time in the reduced GB in comparison with the stoichiometric GB; see Supplementary Note 8 and Supplementary Table 1 for detailed derivation and calculations. The smaller value of *s* parameter (*s*_2_) can result in a larger equilibrium GB order parameter ${\eta }_{{\rm{GB}}(2)}^{{\rm{Equilibrium}}}$ (Fig. 6b). Thus, the DFT calculations quantitatively justify that oxygen reduction can induce a GB disorder-to-order transition.

### AIMD simulations of enhanced GB diffusivities

We further perform large-scale AIMD simulations to calculate and compare the GB diffusivities for the stoichiometric and reduced GBs. Figure 6c shows the calculated GB diffusivities at 840 °C, which are increased markedly in the reduced and ordered GB in comparison with those in stoichiometric and disordered GB. For example, the Bi diffusivity increased by ~5×, Zn diffusivity increased by ~11×, and O diffusivity increased by ~2× in the oxygen reduced and ordered GB (Fig. 6c). Therefore, the AIMD simulations suggest the increased diffusion kinetics of the reduced and ordered GBs to explain the observed increased GB mobilities.

### Charge density maps and Bader charges

The differential charge density maps obtained from DFT calculations further suggest that the increased diffusivity in the reduced GB can be attributed to the weaker charge transfer and chemical bonding (Supplementary Note 9 and Supplementary Fig. 18). Furthermore, we calculate average Bader charges to show that the effective charge on the Bi cations is decreased with the reduction (i.e., one Bi atom losses ~1.4 *e* in the stoichiometric GB vs. ~0.69 *e* in the reduced GB; Supplementary Table 2).

## Discussion

The above thermodynamic model and DFT and AIMD results can be understood intuitively. The presence of aliovalent Bi^3+^ adsorbates (substituting Zn^2+^ cations) in the stoichiometric GB likely promotes interfacial disordering. The oxygen reduction decreases the effective charge on Bi adsorbates to reduce interfacial disordering. These are supported by the calculated differential charge density maps and Bader charges discussed above.

Thus, we can envision the following mechanism. The aliovalent Bi adsorbates serve as charged “hot spots” to provide “pinning” effects at the stoichiometric GB with strong charge transfer (Supplementary Fig. 18) or chemical bonding. In contrast, the oxygen reduction can reduce the effective charge on Bi adsorbates (to weaken the bonding and alleviate “pinning” effects), thereby increasing the kinetics (diffusivities and mobilities) of the reduced GB. Finally, it is worthy to note that the observation of enhanced grain growth in reduced atmospheres (Fig. 4 and Supplementary Fig. 19) further supports our hypothesis that reduction can promote grain growth (even without an applied electric field/current); see Supplementary Note 10 for elaboration.

This work shows that ZnO-Bi_2_O_3_ can be used as a model system to uncover a fundamental mechanism of electrochemically induced GB transitions with significantly increased GB diffusivities, which subsequently result in enhanced and abnormal grain growth. These mechanisms have been further supported by controlled grain growth experiments and large-scale AIMD simulations. A generalized thermodynamic model associated with DFT calculations not only sheds light on the physical origin of GB disorder-to-order transition, but also enables us to forecast other materials in future studies.

These findings have enriched our fundamental understandings of both electric field effects on microstructural evolution and the potentially transformative GB complexion (interfacial phase-like transition) theory via building a bridge between these two areas of great scientific importance and broad technological relevance.

Moreover, electrochemically induced GB transitions can exist in other systems and influence microstructural evolutions and various other properties, with potentially broad technological impacts on a variety of innovative materials processing technologies and electrochemical (or electronic) devices using electric fields and currents. This study also further suggests a new method to tailor the GB structure and properties electrochemically, as well as microstructures (e.g., to intentionally produce graded and far-from-equilibrium microstructures).

## Methods

### Preparation of polycrystal/single crystal/polycrystal (PC/SC/PC) sandwich specimens

First, 0.5 mol% Bi_2_O_3_-doped ZnO powders were prepared by mixing ZnO (99.98% purity, ~18 nm, US Nanomaterials) with bismuth acetate (≥99.99% purity, Sigma Aldrich). Mixed powders were ball-milled for 10 h with a small amount of isopropyl alcohol. Powders were subsequently dried in an oven at 80 °C for 12 h and annealed at 500 °C for 1 h. ZnO $(11\bar{2}0)$ single crystals with both sides polished were purchased from MTI Corporation (Richmond, California, USA). Dense Bi_2_O_3_-doped ZnO PC/SC/PC sandwich specimens were fabricated by spark plasma sintering (SPS) or field-assisted sintering technology at 780 °C for 5 min under a pressure of 50 MPa using a Thermal Technologies 3000 series SPS (Chatsworth, California, USA), and subsequently de-carbonized by annealing at 700 °C for 9 h in air. After sintering, sandwich specimens reached >99% relative densities. Each sandwich specimen was ground to 5.0 × 5.0 × ~1.6 mm^3^ cuboids with a 0.5-mm thick single crystal in between, which completely separated the two polycrystalline regions.

It is worth noting that the maximum solid solubility of Bi_2_O_3_ in the ZnO crystal is <0.06 mol%^28^ so that most added Bi_2_O_3_ (0.5 mol% in the polycrystal) is present at GBs as the liquid-like nanoscale IGFs (see, e.g., Supplementary Fig. 9) or at triple-grain junction as a minor liquid phase at the annealing temperatures of 840–880 °C (above the ZnO-Bi_2_O_3_ eutectic temperature of 740 °C). Nevertheless, we adopt the term “Bi_2_O_3_-doped ZnO” that is commonly used in the ceramics field, albeit we acknowledge the majority of Bi is not doped into the ZnO crystal lattice.

### Annealing with an applied electric current

Dense PC/SC/PC sandwich specimens were sputtered with platinum to form electrodes on both sides of two polycrystalline regions using a Denton Discover 18 Sputter (Moorestown, New Jersey, USA). An external DC electric current was applied on the specimen while annealing at a furnace temperature of 840 °C for 4 h and maintaining a constant DC current density of *J* = 6.4 mA/mm^2^. The specimen temperature, which was higher than the furnace temperature due to the Joule heating, was estimated to be ~865 °C based on the electric power density and radiation heat transfer using a method described previously^22^. The electric field direction was perpendicular to the imbedded ZnO $(11\bar{2}0)$ single crystal. More details of the experimental setup can be found in a prior publication (where the same setup was used for flash sintering experiments)^33^.

The electric potentials and currents were recorded using a high-precision digital multimeter (Tektronix DMM 4050, Beaverton, Oregon, USA) during the experiments. The measured resistance vs. time curve for the PC/SC/PC sandwich can reach steady states after ~50 min. See Supplementary Note 11 and Supplementary Fig. 20.

Following steady-state PC/SC/PC sample, all specimens were air quenched for characterization.

### Annealing experiments without an electric field/current

To rule out the effects of the Joule heating with the applied electric current (that heated the specimen up by ~25 °C), the reference sandwich specimen was annealed at 880 °C for 4 h using the exact same experimental setup but without any external electric field/current (i.e., 40 °C higher in comparison with the furnace temperature of 840 °C for the specimen annealed with the applied electric current). As we noted in the prior section, the specimen temperature was estimated to be ~865 °C based on the Joule heating and radiation heat transfer^22^ for the specimen annealed with the applied electric current. Thus, the annealing temperature of the reference specimen was (intentionally set to be) above the upper limit of the estimated specimen temperature of the case with an applied electric current. This ensures that any increased GB mobilities observed in the specimen with the applied electric current was not due to thermal effects (Joule heating).

The reference PC/SC/PC sandwich specimens were examined before and after the isothermal annealing (without an applied electric field/current), and the relevant SEM cross-sectional images are shown in Supplementary Figs. 6 and 7, respectively.

In addition, three dense polycrystalline specimens were prepared by using the same condition described above, and subsequently annealed at 880 °C for 4 h without electric field/current in air, Ar, and Ar + 5% H_2_, respectively, to investigate the effects of reducing atmospheres on grain growth. The results shown in Fig. 4b–d and Supplementary Fig. 19 further confirm that oxygen reduction can enhance grain growth.

After isothermal annealing, all specimens were air quenched for characterization.

### Characterization of microstructures and grain growth

The densities of sandwich specimens were measured using the Archimedes method. Quenched specimens were characterized by using an ultra-high-resolution scanning electron microscope (Apreo SEM, FEI, Hillsboro, Oregon, USA) on the cross-sections after grinding and polishing. Electron backscatter diffraction (EBSD) mapping was acquired by using an c-wave detector from Oxford Instruments (Concord, MA, USA).

The growth of the single crystal front was measured at 14 locations (×16 measurements per location) along each PC/SC interface for each case. At each location, the migration of the single crystal front (of the PC/SC interface) was averaged from 16 individual measurements with 5 µm intervals. The measured were conducted for both PC/SC interfaces in the specimens annealed with and without an applied electric field (for four cases all together). The results are plotted in Supplementary Fig. 2.

### Photoluminescence spectroscopy

Spatially resolved photoluminescence spectroscopy in the wavelength range from 400 to 700 nm on the cross-sectional surface of the sandwich specimen was carried out on a confocal microscope (Leica SP5, Leica Microsystems, Wetzlar, Germany) equipped with a multiphoton system. The microscope spatial resolution is ~0.4 µm, and the penetration depth is ~50 nm.

### Aberration-corrected electron microscopy and energy dispersive X-ray spectroscopy

Transmission electron microscopy samples were prepared by using a dual-beam focused ion beam/SEM system (Scios, FEI, Hillsboro, Oregon, USA) to lift out specimens of the selected GBs from the cross section based on the EBSD and SEM images.

AC STEM of the GB structures was conducted by using a JEOL JEM-300CF STEM microscope (Akishima, Tokyo, Japan) operating at 300 kV. Both HAADF and bright-field images were recorded.

EDS was used in conjunction with STEM to confirm segregated regions (the GB complexions observed by STEM with bright contrast in HAADF imaging) are Bi-enriched (Supplementary Fig. 13).

### STEM image analyses to reveal order/disorder

To examine the order and disorder (including the partial order in the amorphous-like GBs), we conducted two types of image analyses of the STEM images to reveal the layering vs. lateral orders, as follows.

First, we integrated the STEM HAADF intensities along the direction parallel to the GB to show the layering orders. Two examples of an amorphous-like GB (IGF) vs. an ordered bilayer are plotted as the yellow lines on the top of the STEM images in Fig. 5a, b.

Second, we conducted line-by-line fast Fourier transition (FFT) analysis to probe the lateral periodic orders. Here, we selected a rectangular frame (with the width being equal to the lattice spacing), moved the frame pixel by pixel, and conducted “line-by-line FFT analysis” of the crystalline order. Examples of an amorphous-like GB (IGF) vs. an ordered bilayer are plotted at the bottom panels in Fig. 5a, b.

### First-principles density functional theory (DFT) calculations

The GBGenerator^34^ code in Python Materials Genomics (pymatgen) library^35^ was used to construct ZnO GB structure. The lattice parameters of the ZnO hexagonal structure (*a* = 3.29 Å and *c* = 5.31 Å) were taken from the Materials Project^36^.

An asymmetric GB terminated by $(11\bar{2}0)$ plane (the two GBs shown in Fig. 5a, b) always requires a large simulation cell. Thus, we select the other GB plane to be the non-polar $(10\bar{1}0)$ with a rotate angle of ~53° along the [100] axis to construct a feasible model to mimic the GBs observed in the experiments (Fig. 5) within the limitation of the DFT cell size. Since lattice matching conditions cannot be achieved in three directions to apply periodic boundary conditions, a 15-Å thick layer of vacuum was added to isolate the interaction between two free surfaces created. The final simulation cell is triclinic with parameters: *a* = 0.62 nm, *b* = 1.45 nm, *c* = 5.30 nm, *α* = 104.69°, *β* = 78.69°, and *γ* = 74.70°. This simulation cell contains 240 atoms in total, which is about the largest for effective DFT and AIMD simulations.

The first-principles DFT calculations were performed by using the Vienna ab initio Simulations Package^37,38^. The Kohn–Sham equations were used to solve the projected-augmented wave (PAW) method^39,40^ along with standard PAW potentials for the elements Zn, O, and Bi. The Perdew–Burke–Ernzerhof^40^ exchange-correlation functional was utilized to perform the structural optimization for the GB structure. The lattice parameters of ZnO were kept unchanged during the relaxation and only atomic positions were subjected to relaxation. All atoms were fully relaxed until the Hellmann–Feynman forces were smaller than 0.02 eV/Å. The Brillouin-zone integrations were sampled on a *Γ*-centered 4 × 2 × 1 *k*-point grids. The kinetic energy cutoff for plane waves was set to 400 eV. The convergence criterion for electronic self-consistency was adopted to 10^−4^ eV.

### STEM simulation

STEM HAADF images were simulated by using the QSTEM program^41^. The DFT-optimized GB structures of stoichiometric and reduced Bi-doped ZnO GBs were adopted for imaging simulation. We adopted the electron voltage with a value of 300 kV (same as experiments) for all STEM simulations. The scattering semi-angle for HAADF imaging was set to 60 mrad, the convergence angle was adopted to 20 mrad, and the spherical aberration coefficient was set to 0.5 μm.

### Quantification of GB order/disorder from DFT simulations

A bond-orientational order parameter *η* was calculated for each atom^42^. Subsequently, we defined a dimensionless disorder parameter (${{\eta }}^{{\prime} }\equiv 1-{\eta }$; ${{\eta }}^{{\prime} }$ = 1 for an atom in a liquid and ${{\eta }}^{{\prime} }$ = 0 for an atom in a perfect crystal).

The 1D distribution of disorder parameter ${{\eta }}^{{\prime} }(x)$ was obtained by averaging the ${{\eta }}^{{\prime} }$ values of atoms in the directions parallel to the GB plane. Two examples of disorder parameter profiles ${{\eta }}^{{\prime} }(x)$ for the amorphous-like GB (IGF) vs. the ordered bilayer are shown in Fig. 5g, h. Subsequently, GB excess disorder was quantified by integrating the disorder profile ${{\eta }}^{{\prime} }(x)$. More details can be found in prior publications where similar analyses were performed for atomistic simulations using empirical potentials^9,43,44^ (vs. extending the definitions and methodologies to DFT-relaxed GB structures here in this study).

### Quantification of atomic density profiles from DFT simulations

We used a coarse-grained method to compute 1D atomic density profiles from DFT-relaxed GB structures to compare with the STEM results. In this procedure^9^, a Gaussian function was assigned to each atom and the overall density distribution function can be computed by summing all Gaussian functions. Two examples of Bi profiles for the amorphous-like GB vs. the ordered bilayer are shown in Fig. 5e, f.

### Ab initio molecular dynamics (AIMD) simulations of GB diffusivities

Based on the optimized GB structures, we performed AIMD simulations under the NVT ensemble with a Nose–Hoover thermostat^45,46^ to obtain GB diffusivities. The temperature was set to 1123 K (850 °C), close to the experimental annealing condition. The overall simulation time was set to 1500 fs with a time step of 1 fs. Although the overall simulation time is relatively short (due to the limitation of a very large simulation cell), we believe the GB structures can achieve equilibrium based on monitoring the potential energy vs. time. The *k*-point grids were adopted to 1 × 1 × 1 (*Γ* point only). To avoid the effects of vacuum, we fixed 2~3 monolayer atoms near the free surfaces and only allowed other atoms to move. The atoms’ trajectories during AIMD simulation were used to calculate the mean square displacement (MSD) over time (*t*). Finally. the GB diffusivities for Zn, O, and Bi, respectively, were obtained by linearly fitting the corresponding MSD vs. *t* curves for both stoichiometric and reduced GBs for comparison (Fig. 6c).

## Supplementary information

Supplementary Information

## Data Availability

All data are available from the corresponding authors on reasonable request.
