# Supplementary material for: Discovery of electrochemically induced grain boundary transitions
Source: Nat Commun. 2021 Apr 22;12:2374. doi: 10.1038/s41467-021-22669-0 (PMC8062690; doi:10.1038/s41467-021-22669-0)
Supplement: Supplementary file 1 — Supplementary Information [file 41467_2021_22669_MOESM1_ESM.pdf]

Supplementary Information

Discovery of Electrochemically Induced Grain Boundary Transitions

Jiuyuan Nie [1,#], Chongze Hu [1,2,#], Qizhang Yan [1], and Jian Luo [1,2,*]

[1] Department of Nanoengineering; [2] Program of Materials Science and Engineering
University of California, San Diego
La Jolla, California 92093, U.S.A.

[#] These authors contributed equally.

[*] Corresponding author. E-mail address: jluo@alum.mit.edu (J. Luo).

Table of Contents

Supplementary Notes

1. Discussion of electric effects on microstructural evolution & related innovative processing technologies....3
2. Microstructural evolution of the sandwich specimens (with and without an electric current) 6
3. Defects polarization and the profile of oxygen vacancies in the sandwich specimen 14
4. Analysis of photoluminescence spectra and oxygen vacancy concentrations 16
5. Amorphous-like *vs.* ordered GB structures and EDS confirmation of Bi segregation 19
6. DFT optimized GB structures and comparisons with experiments 25
7. DFT calculations of GB energies and a predicted GB order-disorder transition 29
8. A generalizable thermodynamic model supported by DFT calculations 31
9. Mechanisms of enhanced kinetics in the reduced GB: insights from the DFT calculated differential charge densities and Bader charges 37
10. Enhanced grain growth in reduced atmospheres supporting the proposed mechanism................................40
11. Electric characteristics of the specimen 42

Supplementary Tables:

Table 1. DFT calculations of $s\Delta\theta$ to justify the reduction-induced GB disorder-to-order transition 36
Table 2. Calculated Bader charges for the Bi atoms at the stoichiometric *vs.* reduced GB....................................39

Supplementary Figures

Fig. 1. Schematic illustration of sandwich grain growth experiment with electric field...6
Fig. 2. Measured averaged migration distances with and without electric field 8
Fig. 3. EBSD maps showing enhanced growth at the reduced SC/PC2- interface 9
Fig. 4. EBSD maps showing moderate growth at the oxidized PC1+/SC interface..10
Fig. 5. EBSD maps showing abnormal grain growth in the reduced PC1- region..11
Fig. 6. SEM images of an as-sintered sandwich specimen 12
Fig. 7. SEM images of a sandwich specimen annealed without an applied electric current...................................13
Fig. 8. Band decomposition analysis of photoluminescence spectra 16
Fig. 9. STEM images of disordered GBs in Bi_2O_3-doped ZnO with no electric field 20
Fig. 10. STEM images of disordered GBs in oxidized PC1+ region...21
Fig. 11. STEM images of ordered GBs in reduced PC2- region...22
Fig. 12. Microstructure of abnormal grains near the cathode in reduced PC1- region 23
Fig. 13. EDS analysis confirming GB segregation of Bi 24
Fig. 14. DFT optimized GB structures with different oxygen vacancy concentrations 25
Fig. 15. DFT optimized ordered complexions formed at electrochemically reduced GBs 27
Fig. 16. DFT calculations of the GB energies to predict a GB disorder-order transition...30
Fig. 17. Schematic illustration of GB disorder-to-order transition 33
Fig. 18. Differential charge densities to explain the enhanced diffusion and kinetics in reduced GB 37
Fig. 19. Grain growth of ZnO + 0.5 mol% Bi_2O_3 in air, Ar, and Ar + 5% H_2 40
Fig. 20. Measured specimen resistivity/resistance *vs.* annealing time 42

Supplementary References 43-50

Supplementary Note 1:

Discussion of electric effects on microstructural evolution and related innovative processing technologies

Innovative Sintering Technologies:

Electric fields and currents are used in flash sintering [1,2], electro-sintering [3,4], and field-assisted sintering technology (FAST; commonly known as "spark plasma sintering" or "SPS") [5,6].

First, in the "flash sintering" pioneered by Raj and co-workers [2], ultrafast densification in seconds at reduced furnace temperatures is enabled via directly flowing electric currents through specimens [1,2,7,8]. While recent studies demonstrated that the flash generally initiates as a thermal runaway [9-11] and ultrafast densification stems from the ultrahigh heating rates [12,13], electric fields and currents can significantly influence microstructural evolutions [9,14-21] and induce other unusual phenomena [17,22-25]. Notably, asymmetrical grain growth has been widely observed in flash sintered ZnO [9], ZrO_2 [26], 3YSZ [27], $MgAl_2O_4$ [28], and UO_2 [29], among others [1,17,19], but their underlying mechanisms are largely unknown. Possible roles of grain boundary (GB) complexions on flash sintering have been discussed [12,13,30,31] and explored [7,32], but no in-depth study has been conducted to reveal how they may influence grain growth.

Second, in the "electro-sintering" discovered by Chen and co-workers, an large electric current was used to enhance densification of 8 mol% Y_2O_3-stabilized ZrO_2 (8YSZ) via ionomigration of pores at a low specimen temperature [3,4], which can have drastic impacts on microstructural evolution (to be elaborated subsequently) at the same time [33]. Thus, understanding how electric fields and currents influence the microstructural evolution can not only improve these novel sintering technologies, but also provide new possibilities to control or even tailor microstructures.

Third, electric fields are known to affect the microstructural evolution in the widely used FAST/SPS [19] even if a large portion of the electric current may flow through the graphite tooling surrounding the specimen in the conventional setting (albeit that the electric current can be forced into the specimen via the "flash SPS" setting [34-36]).

A similar effect may also be expected for the most recently reported ultrafast high-temperature sintering (UHS) that can densify ceramics in ~10 seconds [37], where the current is mostly running through the thin carbon heaters around the specimen but the electric field may still affect the microstructural evolution near the specimen surface.

Electrochemical Devices for Energy Storage and Conversion:

Electric fields and currents are present in solid electrolytes used in solid oxide fuel cells [38-40] and solid-state batteries [41], as well as various other electrochemical or electronic devices that use electric fields and currents, where they can cause unexpected (usually undesirable) changes in microstructures. The underlying mechanisms are controversial.

Specifically, fascinating yet often elusive observations of the electric effects of grain growth are briefly discussed using three model systems as exemplars, as follows.

YSZ (Y_2O_3-Stabilized ZrO_2):

Earlier studies already revealed interesting and intriguing observations of the electric field effects on grain growth of YSZ. For example, Conrad *et al.* showed that a relatively weak applied DC or AC field could inhibit grain growth of 3YSZ and attributed it to the interactions of the applied electric fields with space charges [42-46]. In contrast, Chen and colleagues demonstrated that a large applied electric current of ~50 A/cm^2 could enhance the GB mobility by >10 times in the cathode (negative electrode) side discontinuously in 8YSZ [33]. A series of follow-up studies further confirmed the generality of, and investigated, this cathode-side enhanced grain growth phenomenon in several fluorite-type oxides (including 3YSZ) [38,47-49]. Here, Chen and coworkers attributed the enhanced grain growth in the cathode side to the electrochemically driven reduction that lowers the GB migration barriers (from the bulk defects and bulk diffusion point of view, instead of possible GB transitions, which have not yet been examined), and developed a bulk defect chemistry based model [38,47-49]. They further showed that an "oxygen potential transition" in the bulk can induce a phase-like transition behaviors in grain growth [38]. It is yet unknown whether applied electric fields and currents can also change the GB structures to influence the microstructural evolution in YSZ.

Pervoskite $SrTiO_3$:

Rheinheimer *et al.* showed that a weak electric field (with no current, via using "blocking" electrodes) can promote grain growth of $SrTiO_3$ near the negative electrode, which was hypothesized to be resulted from the increased concentrations of oxygen vacancies [50]. However, the exact atomic-level mechanism of how oxygen vacancies can enhance grain growth is unclear. Separate bicrystal experiments by Hughes and van Benthem suggested that an applied electrostatic field (again with no current) could change the structure of (100) symmetric twist GBs in $SrTiO_3$, which was explained from asymmetric "ordering of the oxygen sublattice" in the bicrystals of a special geometric configuration [51,52]. It is unknown whether such structural transitions can also exist at general GBs. In flash-sintered $SrTiO_3$ (with a current), Rheinheimer *et al.* further revealed preferential Ti segregation at general GBs near the positive electrode [53]. However, many open scientific questions remain; a complete understanding of how and why an applied electric field/current can alter the GB structure and/or chemistry and how they subsequently change the grain growth behaviors, as well as the exact underlying mechanisms, have not been established.

ZnO and Bi_2O_3-doped ZnO:

For flash sintering of undoped ZnO in air, our prior study revealed an anode-side abnormal grain growth [9], in contrast to the cathode-side enhanced grain growth observed in YSZ and [33,47-49] and $SrTiO_3$ [50]. This was explained from a hypothesized GB oxidation transition [7]. This hypothesis was supported indirectly by the suppression of this anode-side abnormal grain growth during flash

sintering of pure ZnO in reduced atmospheres [7,54]. However, we could not directly confirm and characterize this hypothesized GB oxidation transition in undoped ZnO (in part because it is difficult, if not impossible, to quench the undoped ZnO specimens to examine the atomic-level GB structures).

In this study, we used Bi_2O_3-doped ZnO (albeit the solid solubility of Bi_2O_3 in the ZnO crystal is <0.06 mol% [55] so that most added Bi_2O_3 is present at GBs or as a secondary phase) to explicitly show, for the first time to our knowledge, that an applied electric current can induce a GB disorder-order transition that can be well quenched for direct characterization by aberration-corrected scanning transmission electron microscopy (AC STEM). Moreover, first-principles calculations confirmed this new mechanism of electrochemical reduction induced transition and *ab initio* molecular dynamics (AIMD) simulations further showed enhanced GB diffusion in reduced and ordered GBs, which fully explained the observed enhanced and abnormal grain growth. Interestingly, enhanced grain growth occurred in the reduced region in Bi_2O_3-doped ZnO near the cathode, in contrast that in the oxidized region near the anode for undoped ZnO [9]. Here, the Bi_2O_3 segregation at GBs enabled us to better quench the interfacial structures for detailed characterization and subsequent modeling, thereby establishing a complete link of the underlying mechanisms of how an applied electric field induce a GB structural transition to lead to enhanced grain growth, for the first time in this study. Moreover, a generalizable thermodynamic model has been established for the reduction induced GB disorder-order transitions.

Supplementary Note 2:
Microstructural evolution of the sandwich specimens (with and without an applied electric current)

The Polycrystal 1/Single Crystal/Polycrystal 2 (PC1/SC/PC2) specimen used in the grain growth experiment with a constant applied electric current is schematically shown in Supplementary Fig. 1 below.

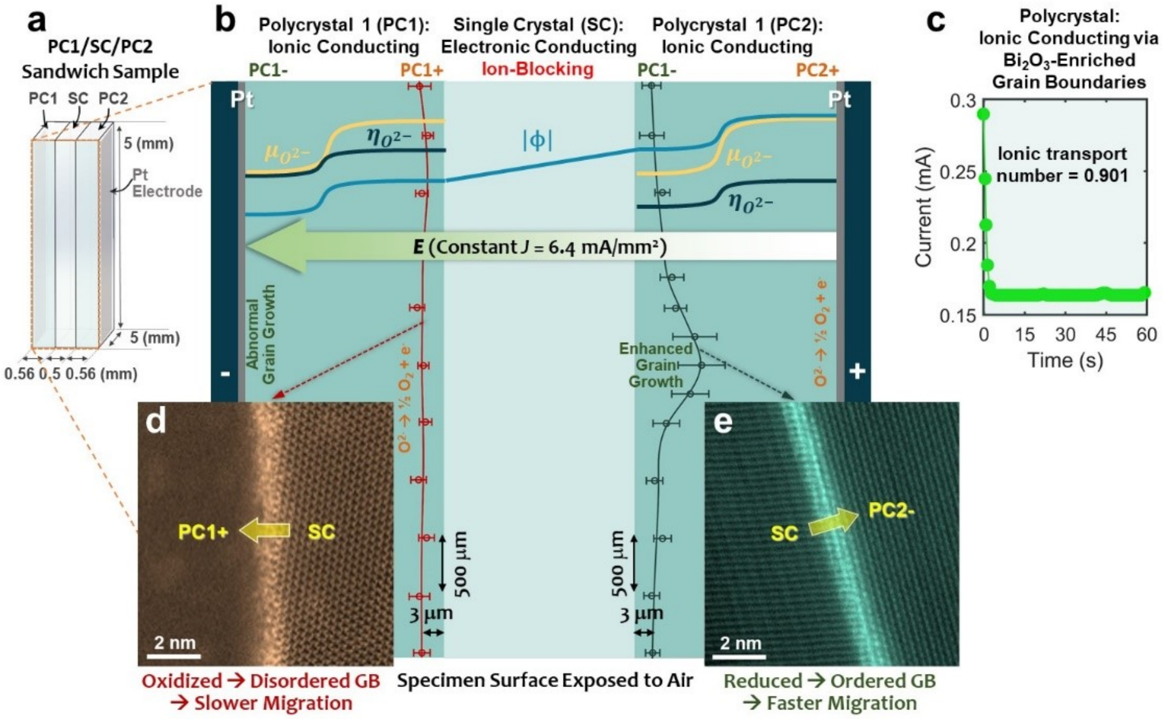

Supplementary Fig. 1. The schematic illustration of the sandwich grain growth experiment with a constant applied electric current. a, Schematic drawing of the sandwich sample. **b**, Proposed profiles of electric (ϕ), chemical ($\mu_{O^{2-}}$), and electrochemical ($\eta_{O^{2-}}$) potentials *vs.* locations. The measured migration distances at PC1+/SC and SC/PC2- regions are plotted. Noting that the aspect ratios are not drawn to the scale. Grain boundaries (GBs) in the two polycrystals are mostly ion-conducting via the Bi_2O_3-enriched liquid-like intergranular films (IGFs), while the ZnO single crystal is electron-conducting but ion-blocking. **c**, The ionic transport number measurement of a ZnO + 0.5 mol% Bi_2O_3 polycrystalline specimen at 840 °C. The measured high ionic transport number of ~0.9 indicates that the specimen is mostly ion-conducting (via the Bi_2O_3-enriched liquid-like IGFs). Consequently, PC1- and PC2- regions must be reduced, while PC1+ and PC2+ regions must be oxidized. See Supplementary Note 3 for detailed discussion. Representative STEM HAADF images of (**d**) a slow-moving disordered GB at the oxidized PC1+/SC interface and (**e**) a fast-moving ordered GB at the reduced SC/PC2- interface.

The key observations of the "unusual" microstructural evolutions in the PC1/SC/PC2 specimen induced by an applied electric current are summarized as follows:

(1) The PC1+/SC interface, which was oxidized (as indicated by the generation of pores presumably due to the oxidation reaction that produced O_2 gas: $O^{2-} \rightarrow \frac{1}{2}O_2 + e^-$), only had moderate migration of ~3 μm (Fig. 2d, e and Supplementary Fig. 4).

(2) In contrast, abruptly enhanced migration of the SC/PC2- interface was observed (Fig. 2f, g

and Supplementary Fig. 3) in the reduced middle section of the SC/PC2- interface (to a maximum of ~10 μm at the center, which significantly increased GB mobility in comparison with both the oxidized PC1+/SC interface and the two PC/SC interfaces in the reference specimen without an applied electric field/current, as elaborated subsequently).

(3) Abnormal grain growth took place in the reduced PC1- region near the negative electrode (cathode) that resulted in extremely large grains, as clearly evident in the SEM images and corresponding EBSD maps in Fig. 2b, c and Supplementary Fig. 5.

To better quantify the grain growth, two reference specimens were fabricated and characterized (both with symmetric grain growth without an applied electric field/current), including:

(i) An as-sintered PC/SC/PC sandwich specimen (prior to annealing at 880 °C), where the averaged migration distances at two PC/SC interfaces were measured to be 2.81 ± 0.94 μm and 2.72 ± 0.88 μm, respectively (Supplementary Fig. 6).

(ii) Another reference PC/SC/PC sandwich specimen annealed at 880 °C for 4 hours without an external electric field/current, where the averaged migration distances at two PC/SC interfaces were measured to be 3.63 ± 0.91 μm and 3.60 ± 0.79 μm, respectively (Supplementary Fig. 7).

Supplementary Fig. 2 shows a quantitative comparison of the PC1/SC/PC2 specimen annealed with an applied electric current at a furnace temperature of 840 °C (with an estimated specimen temperature of ~865 °C considering the moderate Joule heating) for 4 hours and the reference specimen (discussed in Case (*ii*) above) that was annealed without an applied electric field/current at 880 °C (intentionally set to be slightly higher that the upper limit of specimen temperature in the case with an applied electric current) for 4 hours. A critical comparison of quantitative measurements of the migration distances *vs.* lateral locations of four SC/PC interfaces is shown in Supplementary Fig. 2 and discussed as follows:

(A) The migration distances of the oxidized SC/PC1- interface of the specimen annealed with an applied electric current were measured to be 3.04 ± 0.82 μm (*i.e.*, slightly lower than the ~3.6 μm in the reference specimen without an applied electric current).

(B) The migration distances of the PC1+/SC interface of the specimen annealed with an applied electric current were significantly increased in the middle (mostly reduced) section of the specimen to a maximum of ~10 μm growth at the center. The enhanced growth occurred abruptly in the middle (~10-15% of the specimen width, forming a ~500 μm wide protrusion), more reduced region; it decayed to normal growth distances of ~3-4 μm near the (less reduced) free surfaces that were exposed to air, as shown in Supplementary Fig. 2

In summary, the increased GB mobilities occurred in the reduced PC1- and PC2- regions, while the grain growth was moderate in the oxidized region, on a par with that in the reference specimen without an applied electric field/current. Quantitative measurements and a careful comparison have clearly demonstrated that an applied electric current can abruptly increase GB mobility in the electrochemically reduced regions.

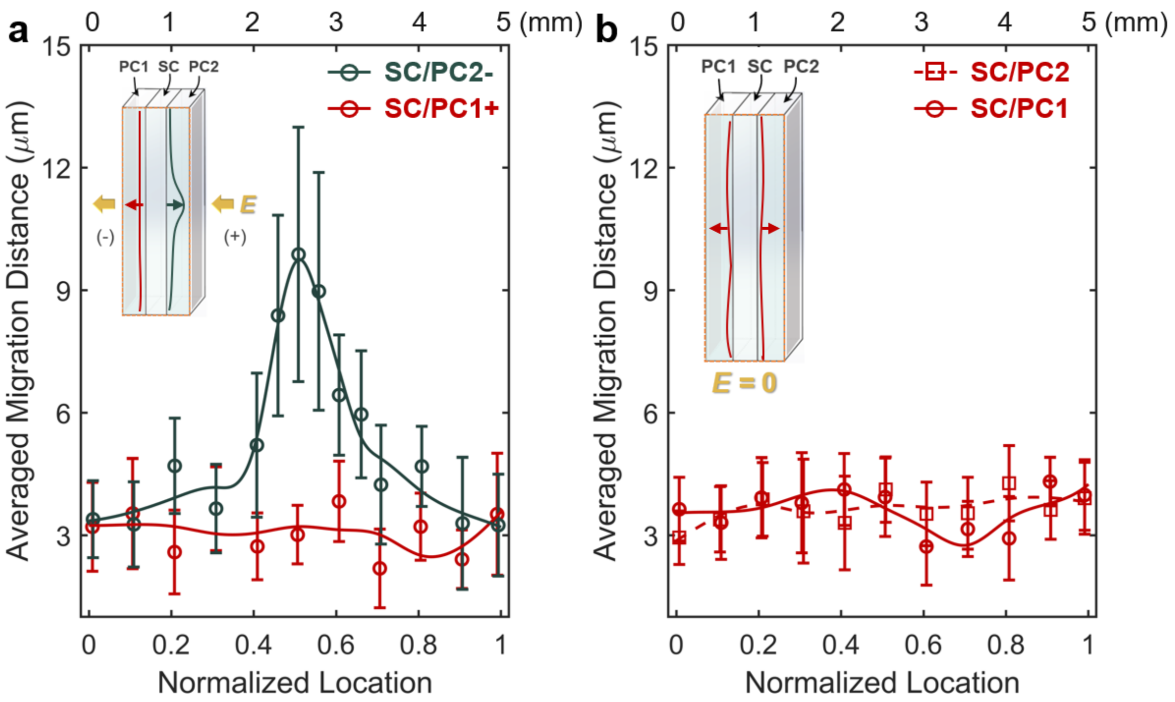

Supplementary Fig. 2. Measured averaged migration distances of the single-crystal fronts *vs.* normalized location (to a 5-mm total length) of Bi_2O_3-doped ZnO PC1/SC/PC2 sandwich specimens annealed with and without an applied electric current. The migration distances the single crystal fronts at both sides were measured from cross-sectional SEM images at the two SC/PC interfaces in (**a**) one sandwich specimen annealed at a furnace temperature of 840 °C with a constant current density of J = 6.4 mA/mm^2 (with an estimated specimen temperature ~865 °C considering the moderate Joule heating) and (**b**) another reference sandwich specimen annealed at 880 °C without electric field/current (E = 0). Both specimens were annealed for 4 hours and subsequently quenched. Each data point was averaged from 16 individual measurements with 5 μm intervals (with the standard deviation as the error bar). Abruptly enhanced growth was observed in the intermediate (*i.e.*, more isolated and reduced) section of the SC/PC2- interface in the specimen annealed with an applied electric current. It is noted that the annealing temperature (880 °C) of the reference specimen was intentionally set to be slightly higher than the estimated specimen temperature (of ~865 °C) for the case with the applied electric current to unequivocally demonstrate that the observed enhanced migration of the SC/PC2- interface is not a temperature (Joule heating) effect.

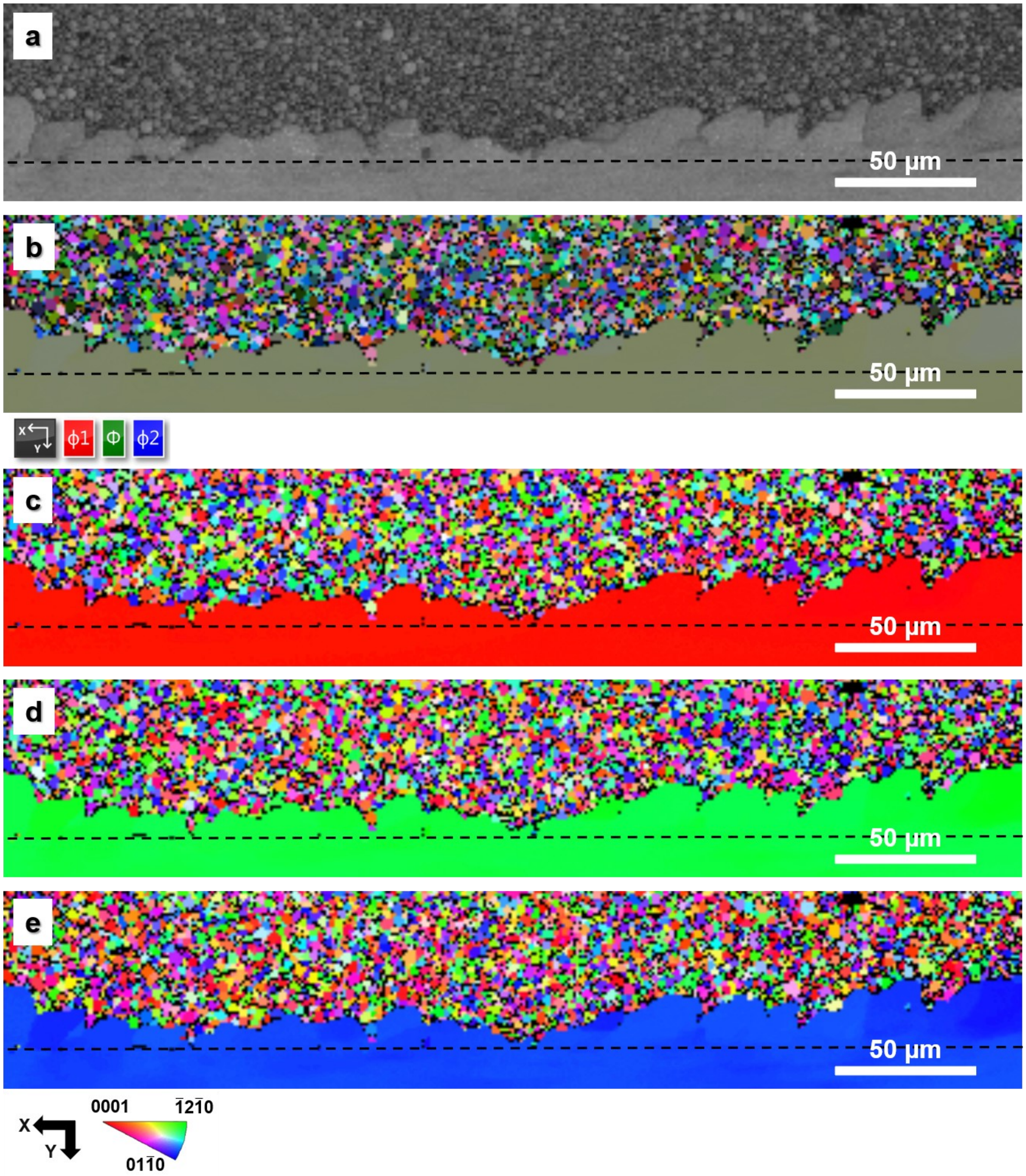

Supplementary Fig. 3. EBSD mapping of the reduced SC/PC2- region with enhanced growth in the Bi_2O_3-doped ZnO sandwich specimen annealed with a constant electric current density of $J = 6.4$ mA/mm^2. **a**, Band contrast of the EBSD map, which shows Kikuchi bands. **b**, Euler maps of the SC/PC2- interface, showing the enhanced growth along the original single crystal orientation. **c-e**, The X, Y, and Z direction inverse pole figure (IPF) orientation maps. The color scheme and reference orientations are shown in the legends.

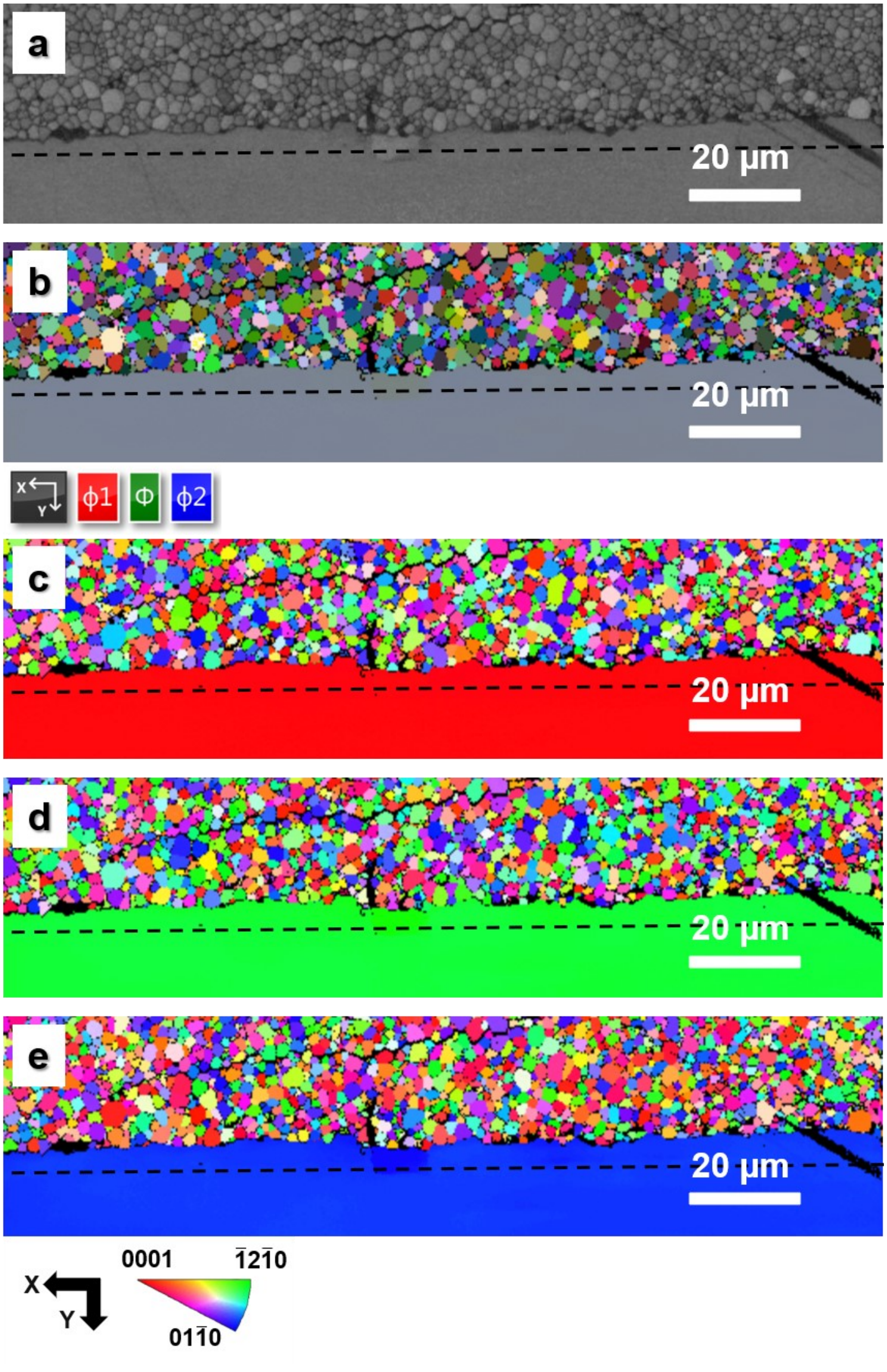

Supplementary Fig. 4. EBSD mapping of the oxidized SC/PC1+ region without enhanced growth in the Bi_2O_3-doped ZnO sandwich specimen annealed with a constant electric current density of J = 6.4 mA/mm^2. **a**, Band contrast of the EBSD map, which shows Kikuchi bands. **b**, Euler maps of the SC/PC1+ interface, showing the growth along the original single crystal orientation. **c-e**, The X, Y, and Z direction inverse pole figure orientation maps. The color scheme and reference orientations are shown in the legends.

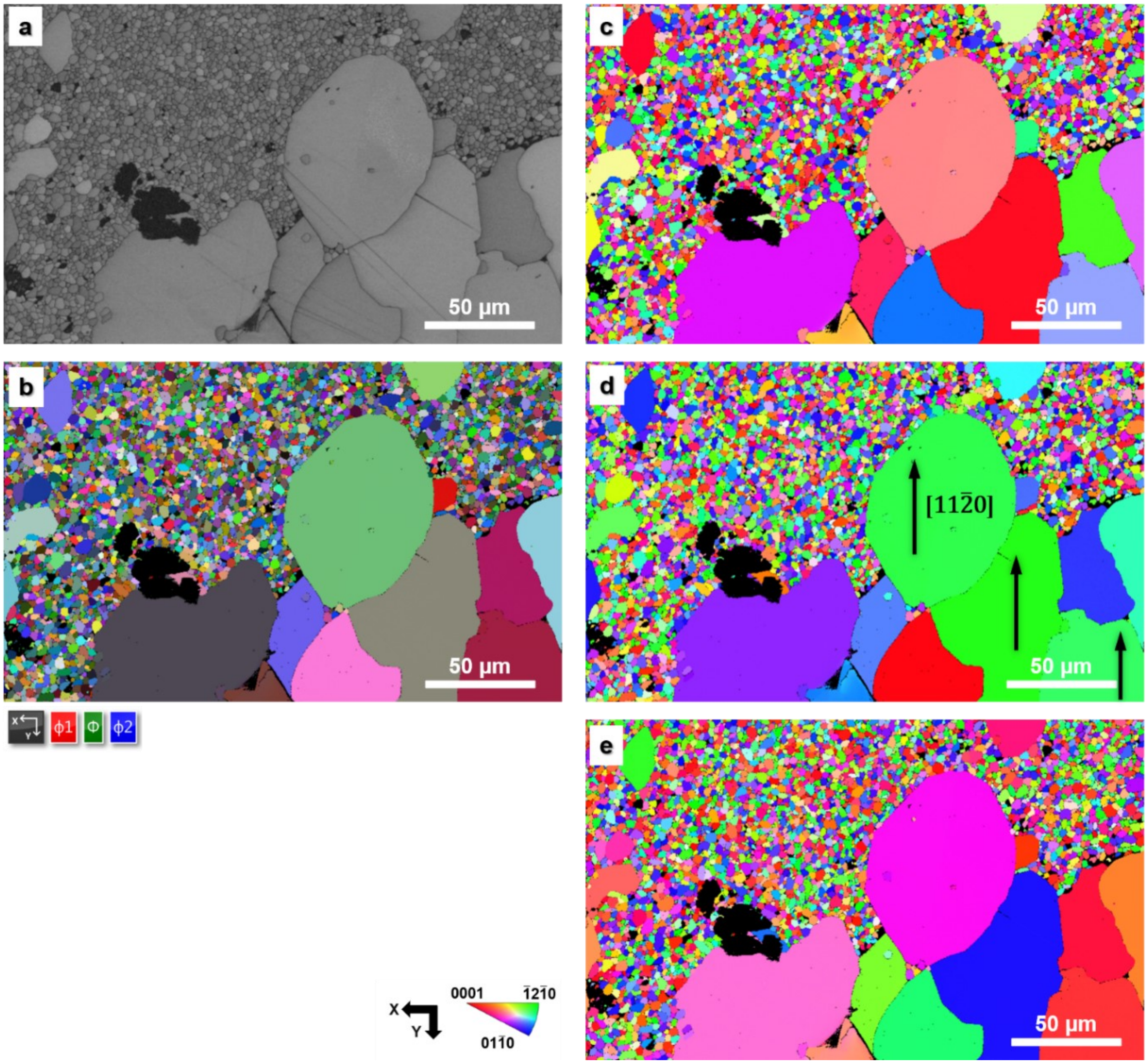

Supplementary Fig. 5. EBSD mapping of the abnormal grains near the cathode side (in the electrochemically reduced PC1- region) in the Bi_2O_3-doped ZnO sandwich specimen annealed with a constant electric current density of J = 6.4 mA/mm^2. a, Band contrast of the EBSD map, which shows Kikuchi bands. **b**, Euler maps of the PC1- region, showing the abnormal grain growth. **c-e**, The X, Y, and Z direction inverse pole figure (IPF) orientation maps. The color scheme and reference orientations are shown in the legends. A preferential crystallographic orientation, [11$\bar{2}$0], is identified in Panel (d) Y-IPF.

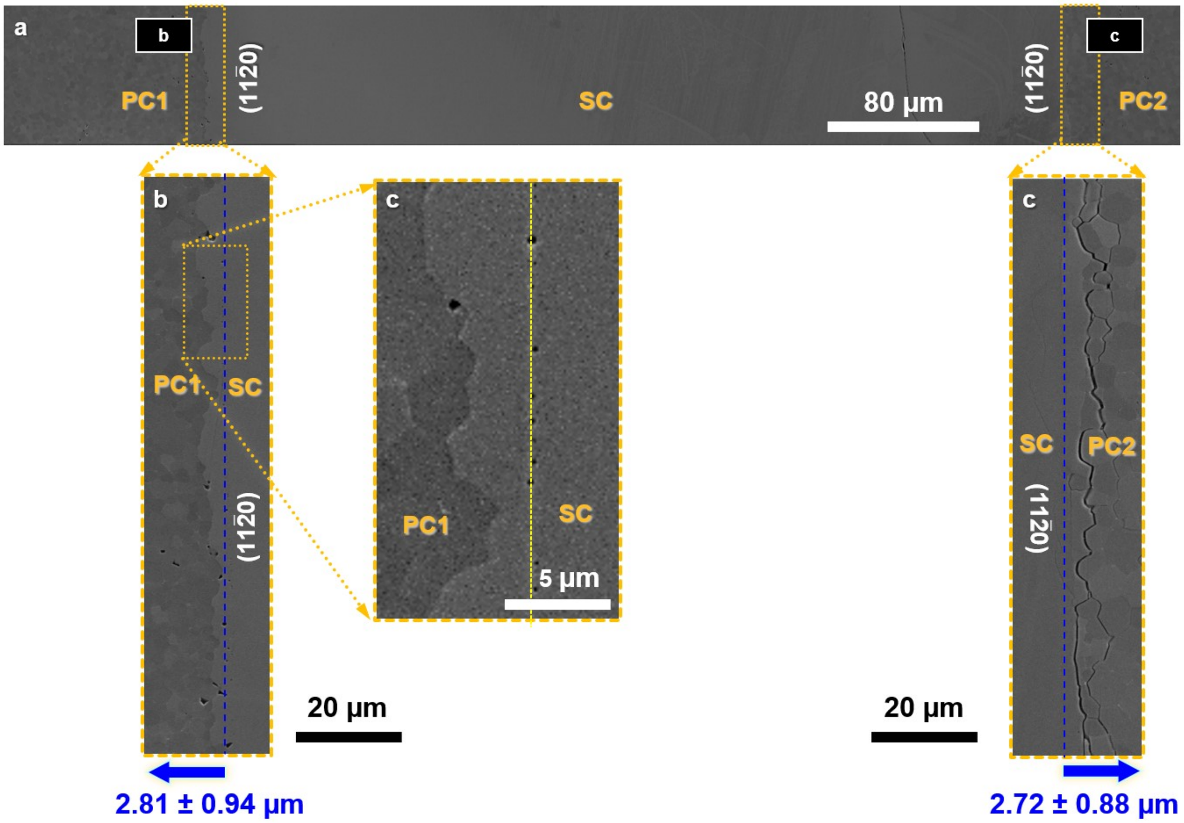

Supplementary Fig. 6. Microstructure of an as-sintered Polycrystal 1/Single Crystal/Polycrystal 2 (PC1/SC/PC2) sandwich specimen. a, Cross-sectional SEM micrograph of this Bi_2O_3-doped ZnO PC1/SC/PC2 sandwich specimen densified using SPS at 780 °C for 5 minutes under 50 MPa pressure, followed by de-carbonization annealing in air at 700 °C for 9 hours. **b**, **c**, High magnification SEM micrographs of the SC/PC interfaces at both sides. Aligned holes were used to identify the locations of the original surfaces of the single crystal, as shown in an enlarged image in Panel (c). The migration distances of the SC fronts on both sides are similar, which measured to be ~2.7-2.8 µm on average.

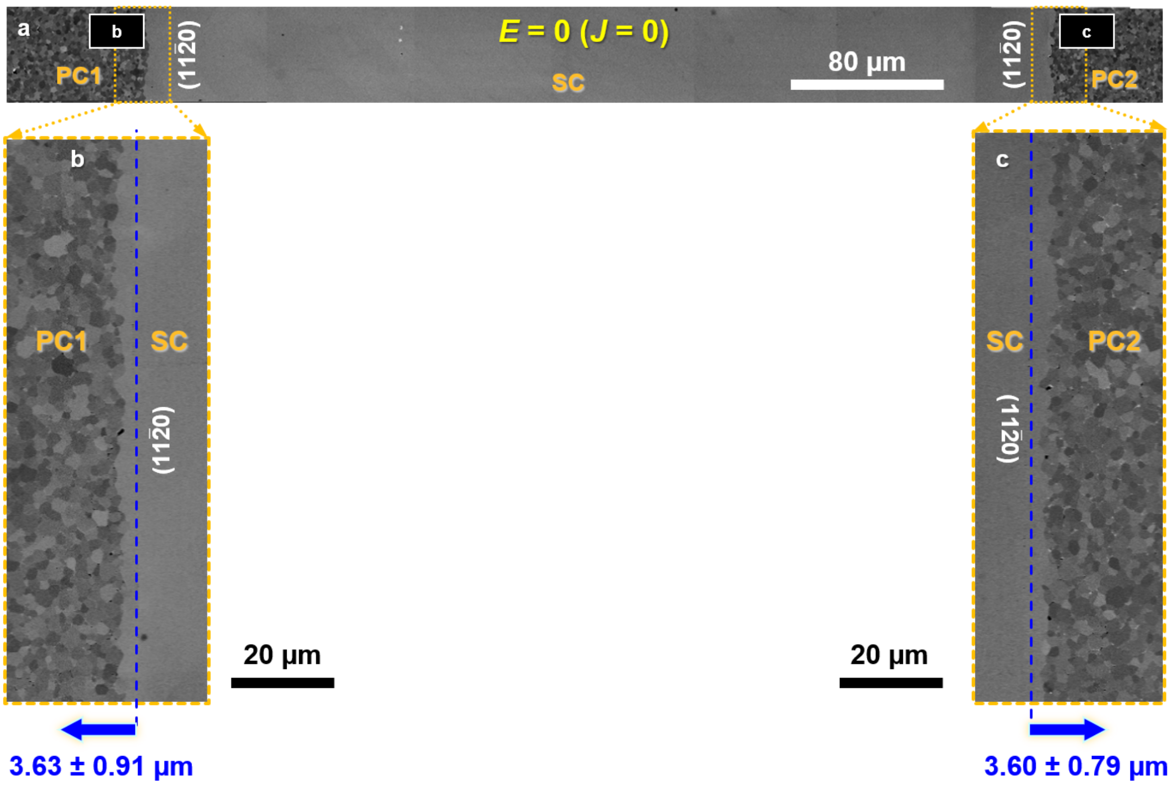

Supplementary Fig. 7. Microstructure of a reference Bi_2O_3-doped ZnO PC1/SC/PC2 sandwich specimen annealed without an electric field isothermally at 880 °C for 4 hours and subsequently quenched. a, Cross-sectional SEM micrograph of this PC1/SC/PC2 specimen. **b**, **c**, High-magnification SEM micrographs of the SC1/PC and SC/PC2 interfaces. Blue dashed lines indicate the original interfaces. The migration distances of the single crystal fronts on both sides are similar, which measured to be ~3.6 µm on average. Note that the migration distances here include the growths of the single crystal during the isothermal annealing as well as in prior fabrication steps (~2.7-2.8 µm on average during SPS and decarbonization annealing, as shown in Supplementary Fig. 6).

Supplementary Note 3:

Defects polarization and the profile of oxygen vacancies in the sandwich specimen

The Fig. 2h schematically illustrates the Polycrystal 1/Single Crystal/Polycrystal 2 (PC1/SC/PC2) specimen, where with an applied current created the reduced PC1- and PC2- and oxidized PC1+ and PC2+ regions.

It is well known that ZnO single crystal is an electronic conductor (semiconductor) [56], but Bi_2O_3 is an oxygen ionic conductor [57]. Interestingly, the Bi_2O_3-enriched GBs (liquid-like IGFs) in Bi_2O_3-doped ZnO can be ion-conducting. To prove its ionic conductivity, our measurement showed a high ionic transfer number of ~0.9 in the polycrystalline Bi_2O_3-doped ZnO at 840 °C; see Supplementary Fig. 1c, which is also shown below for quick access.

Thus, the two PC regions in the PC1/SC/PC2 sandwich specimen are mostly oxygen ion conducting, separated by the electron-conducting but ion-blocking SC. Thus, an applied electric current in the PC/SC/PC should create two reduced regions PC1- and PC2- and two oxidized regions PC1+ and PC2+ with discontinuous oxygen chemical potentials at the PC1/SC and SC/PC2 interfaces, as schematically shown in Fig. 2h.

- On the one hand, the formation of the reduced regions with increased concentration of oxygen vacancies was proved directly by photoluminance spectroscopy (discussed in Supplementary Note 4 subsequently).
- On the other hand, the presence of the oxidized regions was indicated by the generation of pores (with the oxidation reaction: $O^{2-} \rightarrow ½O_2 + e^-$) as evident, *e.g.*, the SC/PC1+ region in Fig. 2d (which are provided below for quick access) and the PC2+ region (as shown in the SEM image on the right side below).

Here, we can use a model to explain the defects polarization and the profile of oxygen vacancies shown in and Fig. 2h. Let us assume oxygen vacancies ($V_O^{\bullet\bullet}$ in the Kröger-Vink notation) as the main defects (similar results can be extended to other types of charged defects). On the one hand, the carriers with positive charge ($V_O^{\bullet\bullet}$) will move along the direction of the electric field and accumulate at the SC/PC2- interface (since the SC is ion blocking) as well as PC1- region near the cathode (blocking Pt electrode), thereby forming two locally reduced regions (Fig. 2h). On the other hand, PC1+ and PC2+ regions should remain oxidized (mostly stoichiometric, with minimum oxygen vacancies), where the oxidation reaction ($O^{2-} \rightarrow ½O_2 + e^-$) occurs as evident by the formation of porosity (see, *e.g.*, Fig. 2d).

Subsequently, we can plot the schematic profiles of electric potential (ϕ), chemical potential ($\mu_{O^{2-}}$), and electrochemical potential ($\eta_{O^{2-}}$) across the sandwich specimen in Fig. 2h, following a similar model from prior electrochemical studies of solid-oxide fuel cells (SOFCs) [38]. Different from the typical SOFC setups [38], our sandwich specimen was annealed in air (both sides) so that schematic profiles shown in Fig. 2h represent the middle part of the specimen (in the cross-

sectional direction perpendicular to the applied electric field/current); consistently, there was more reduction in the middle section of the PC2- region that increased GB mobility, as shown in Supplementary Note 2.

Notably, the chemical potential $\mu_{O^{2-}}$ decreases nonlinearly along the direction of electric field in the polycrystal regions, with an abrupt jump (so called "oxygen potential transition" demonstrated by Chen and co-workers [38], a character of a mixed electron-ion conductor). In addition, the electric potential ϕ should also change abruptly in the polycrystals (as shown in the model proposed by Chen and co-workers [38]), but transit continuously at the PC/SC interfaces. Specifically, these profiles should be determined by applying the Wagner-type transport theory [58]:

$$\frac{d\phi}{dx} = -\frac{i_{tot}}{\sigma_{tot}} + \frac{t_{O^{2-}}}{2e}\frac{d\mu_{O^{2-}}}{dx} + \frac{t_{e^-}}{e}\frac{d\mu_{e^-}}{dx}, \quad (1)$$

where i_{tot} is the total current density (flux), σ_{tot} is the total (ionic plus electronic) conductivity, e is the charge of electron, and $t_{O^{2-}}$ and t_{e^-} are the transport numbers of oxygen ions and electrons, respectively. The electrochemical potential is given by:

$$\eta_{O^{2-}} = \mu_{O^{2-}} - 2e\phi \quad (2)$$

To solve equation (1), we may assume that (*i*) local chemical equilibria in the PC1+ and PC2+ regions with the reaction: $O^{2-} = \frac{1}{2}O_2 + 2e^-$, and (*ii*) $\Delta\mu_e$ equals to 0 with the blocking Pt electrodes. Then, the electrochemical potential of O^{2-}can be obtained:

$$\frac{\partial\eta_{O^{2-}}}{\partial x} = \frac{2ei_{tot.}}{\sigma_{tot.}} + (1 - t_O)\frac{\partial\mu_{O^{2-}}}{\partial x} \quad (3)$$

Based on equations (1-3) and the above assessments, the electric, chemical, and electrochemical potential profiles can be schematically plotted in Fig. 2h. Unfortunately, we cannot quantify these profiles because most of the parameters used in equations (1-3) are unknown. Yet, it can be concluded that an applied current through the PC1/SC/PC2 sandwich would create the reduced PC1- and PC2- and oxidized PC1+ and PC2+ regions because the SC is ion-blocking and the PC1 and PC2 regions are ion-conducting (with a measured ionic transfer number of ~0.9, as shown in Supplementary Fig. 1c).

Supplementary Note 4:

Analysis of photoluminescence spectra and oxygen vacancy concentrations

Photoluminescence spectroscopy was used to analyze oxygen defects in the ZnO sandwich specimen induced by an applied electric current (Fig. 3).

A typical photoluminescence spectrum of ZnO has two main peaks associated with the oxygen defects in the (*i*) UV emission (at ~3.25 eV) due to exciton-bound states and (*ii*) visible light emission (at ~2.15 eV) due to deep-level states [59,60]. Since oxygen vacancies act as deep-donor defects [61], we collected photoluminescence signals in the visible light region from 400 to 700 nm (see, *e.g.*, Supplementary Fig. 8b, c).

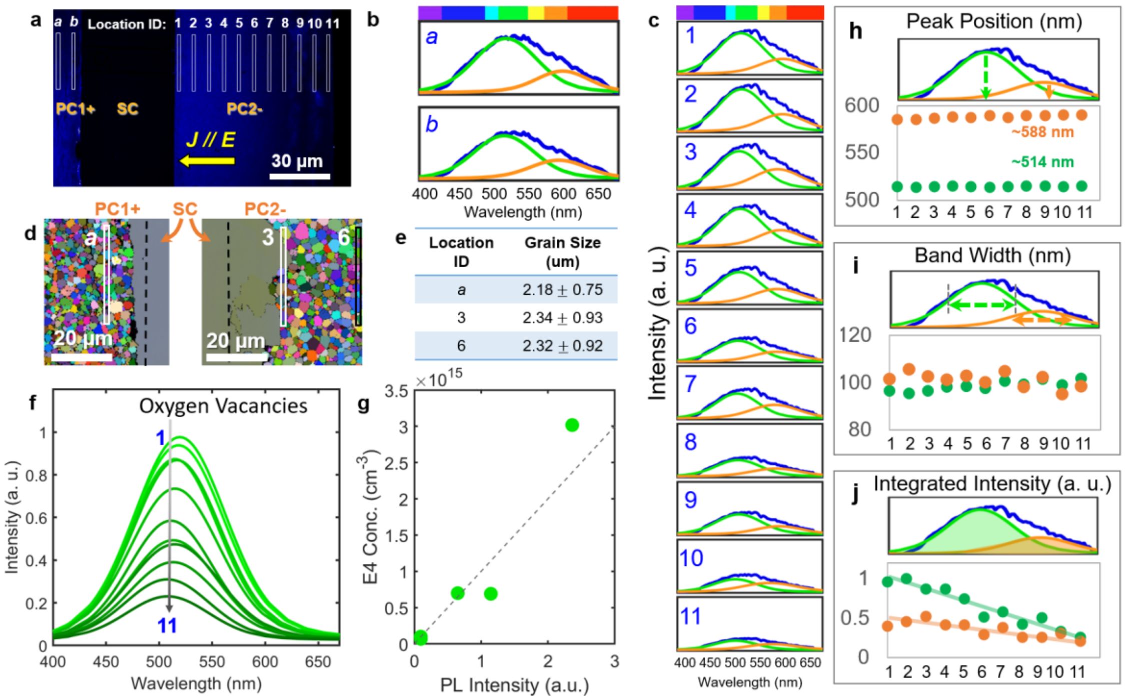

Supplementary Fig. 8. Band decomposition and analysis of the photoluminescence spectra of the Bi_2O_3-doped ZnO PC1/SC/PC2 sandwich specimen annealed with a constant applied electric current, showing the enrichment of oxygen defects in the reduced PC2- region. a, Photoluminescence intensity maps at the 526 nm wavelength of the cross-sectional PC1/SC/PC2 specimen. **b**, Band decomposition of photoluminescence spectra in the PC1+ region collected at Locations *a* and *b* labeled in Panel (a). **c**, Band decomposition of photoluminescence spectra in the PC2- region collected at Locations 1-11 labeled in Panel (a). The photoluminescence bands can be decomposed to two overlapping emission bands. The "green" band in Panel (b) and (c) is related to the emission from oxygen vacancies[61]. **d**, The EBSD maps of the PC1+ and PC2- regions. **e**, Measured grain sizes of polycrystals near the interfaces with the single crystal (at Location *a* in the PC1+ region and Location 3 in the PC2- region), as well as at Location 6 in the PC2 away from the SC/PC2- interface, showing no substantial differences in the grain sizes; thus, the observed variations in the photoluminescence intensities are not due to the grain size effects. **f**, The evolution of the green-emission bands (representing oxygen vacancies) from Location 1 to 11 (away from the SC/PC2- interface). **g**, The concentration of the so-called "E4 defects" (corresponding to the 4th energy level below the conduction band; attributed to oxygen vacancies based on DFT calculations[61-63]) *vs*. the green-emission band intensity at 2.45 eV (~506 nm) measured by deep level transient spectroscopy (DLTS)[61]. The decreasing of green-emission band intensity from Location 1 to 11, as shown in Panel (f), indicates decreasing oxygen vacancy concentrations. **h**-**j**, The peak position, band width, and integrated intensity of the separated "green" and "orange" bands at Locations 1-11.

Subsequently, we decomposed photoluminescence spectra into "green" (centered at ~510 nm) and "orange" (centered at ~600 nm) bands to further analyze the defects, particularly the distribution of oxygen vacancies. It is worth noting that the two decomposed bands are named as "green" and "orange" because their center wavelengths are in the green and orange spectra, respectively, albeit that both peaks are broad and overlap significantly. It is well established from prior studies that the presence of oxygen vacancies dominantly contribute to the green-band emission around 510 nm [59,61,64,65]. Most prior studies considered orange-band emission to be related to zinc interstitials [65,66], albeit a debate [61].

In this study, we focused on the decomposed "green" bands to probe oxygen vacancy distribution. Hoffman *et al.* used deep level transient spectroscopy (DLTS) to find a so-called "E4 defect level" that is ~0.53 eV below conduction band (CB), similar to the defect level of the oxygen vacancy (*i.e.*, ~0.6 eV below CB based on first-principles calculations); thus, they attributed this "E4 defect" (*a.k.a.* the 4th defect energy level below the CB) to oxygen vacancy [61]. They also found that the concentration of "E4 defects" (oxygen vacancies) is proportional to the photoluminescence intensity (Supplementary Fig. 8) so that the green-band intensity should have a positive correlation with the oxygen vacancy concentration [61]. Thus, we used the intensity of this decomposed green band to probe the distribution of oxygen vacancies in this study.

To quantify the intensity of the green-band emission, we used the CasaXPS software to fit decomposed bands by using the asymmetric Lorentzian lineshape method with Gaussian distribution. The lineshape parameters were set to the default values: $\alpha = 1.53$ and $\beta = 243$. The band positions were constrained in the range of 415 to 670 nm.

The decomposed green and orange bands are plotted for Locations *a* and *b* in Supplementary Fig. 8a, b and for Locations 1-11 in Supplementary Fig. 8c. The fitted peak positions and band widths for the two bands remain mostly constant (Supplementary Fig. 8h, i), while the integrated intensity of the green band, which represents in the oxygen vacancy concentration as discussed above, decreases from Location 1 to Location 11 (*i.e.*, moving away from the SC/PC2- interface), as shown in Supplementary Fig. 8j, f. The intensity of the green band is also lower at the PC1+ region (Location *a*), as shown in Fig. 3e. This suggests a higher concentration of oxygen vacancies near the SC/PC2- interface.

The photoluminescence emission peak was not observed in the single crystal (SC) region, thereby suggesting the defects are mostly present at GBs. It is in fact well known that photoluminescence peaks associated with oxygen defects are mostly from GBs in ZnO, so that the grain size may also have an effect on photoluminescence intensity [59,64]. Here, we need to exclude this effect to draw a rigorous conclusion. Our measurements based on EBSD maps show the reduced PC2- region actually has slightly larger mean grain size (2.34 ± 0.93 μm) than the oxidized PC1+ (2.18 ± 0.75 μm) and the grain size does not change significantly (virtually a constant within ~50 μm) in the PC2- region (*e.g.*, 2.34 ± 0.93 μm at Location 3 and 2.32 ± 0.92 μm at Location 6), as shown in Supplementary Fig. 8e. Thus, the decreasing intensity of the green band away from

the SC/PC2- interface in the PC2- region (Supplementary Fig. 8f), as well as the higher intensity in the (reduced) PC2- region than that in the (oxidized) PC1+ region (Fig. 3c), is not due to the grain size effect. Instead, the PL analysis directly showed a higher concentration of oxygen vacancies in the reduced PC2- region, particularly near the SC/PC2- interface.

Notably, the intensity of the green-band emission in the PC2- region is higher than that in the PC1+ region (as shown in Fig. 3e). This directly revealed the presence of more oxygen vacancies in the reduced SC/PC2- interface (*i.e.*, the electrochemically reduced region where GB disorder-to-order transition led to abruptly increased GB mobilities) than the oxidized PC1+/SC interface (with the disordered and slow-moving GBs).

Supplementary Note 5:

Amorphous-like *vs.* ordered GB structures and EDS confirmation of Bi segregation

Amorphous-like *vs.* Ordered GB Structures: Selected representative AC STEM high-angle annular dark-field (HAADF) and bright field (BF) images shown in Fig. 1 suggest that electrochemical reduction induced a GB disorder-to-order transition, which subsequently led to abruptly increased GB mobilities.

Key observations of slow-moving disordered GBs vs. fast-moving ordered GBs in four cases are summarized as follows:

- Amorphous-like GBs, commonly known as intergranular "glassy" films (IGFs, albeit the existence of partial orders [68,69]), have been observed in the reference specimen without an applied electric field/current (Supplementary Fig. 9 and Fig. 1a, b).
    - Such nanometer-thick IGFs, which are ubiquitously observed in sintered ceramics [70], can alternatively be understood to be liquid-like interfacial films that adopt an "equilibrium" thickness on the order of 1 nm in response to a balance of attractive and repulsive interfacial interactions (originally proposed by Clarke [71]) or disordered multilayer adsorbates (originally proposed by Cannon *et al.* [72]).
    - Specifically, such nanoscale liquid-like IGFs have been observed to form at all general GBs (examined to date) in Bi_2O_3-sataured ZnO at thermodynamic equilibria both above and below the bulk eutectic temperature (740 °C), as well as some un-saturated specimens below the bulk solid solubility limits [67,73,74].
- Amorphous-like (disordered) GBs or nanoscale IGFs of similar character have also been observed at the oxidized, slow-moving PC1+/SC interface (Supplementary Fig. 10 and Fig. 1e, f) in the specimen with an applied electric current, which exhibit a similar mobility as the PC/SC interfaces in the reference specimen without an applied electric field.
- In contrast, ordered GB complexions (ordered Bi adsorbates) have been observed at the reduced, fast-moving SC/PC2- interface (Supplementary Fig. 11 and Fig. 1g, h) in the specimen with an applied electric current.
- In addition, ordered Bi adsorbates have also been observed at the GBs of the fast-moving abnormal grains in the reduced PC1- region (Supplementary Fig. 12 and Fig. 1c-d) in the specimen with an applied electric current.

In summary, the slow-moving GBs in the reference specimen without an applied electric field/current and in the oxidized region of the specimen with an applied electric current are all disordered (forming nanoscale amorphous-like or liquid-like IGFs), while the fast-moving GBs in electrochemically reduced regions are all ordered (albeit different ordered structures, which are presumably due to the different crystallographic GB characters of at different general GBs randomly selected from the specimen). Representative AC STEM images are shown in Fig. 1; additional images and cases are documented in Supplementary Figs. 9-12 show the generality of the observations slow-moving disordered GBs *vs.* fast-moving ordered GBs.

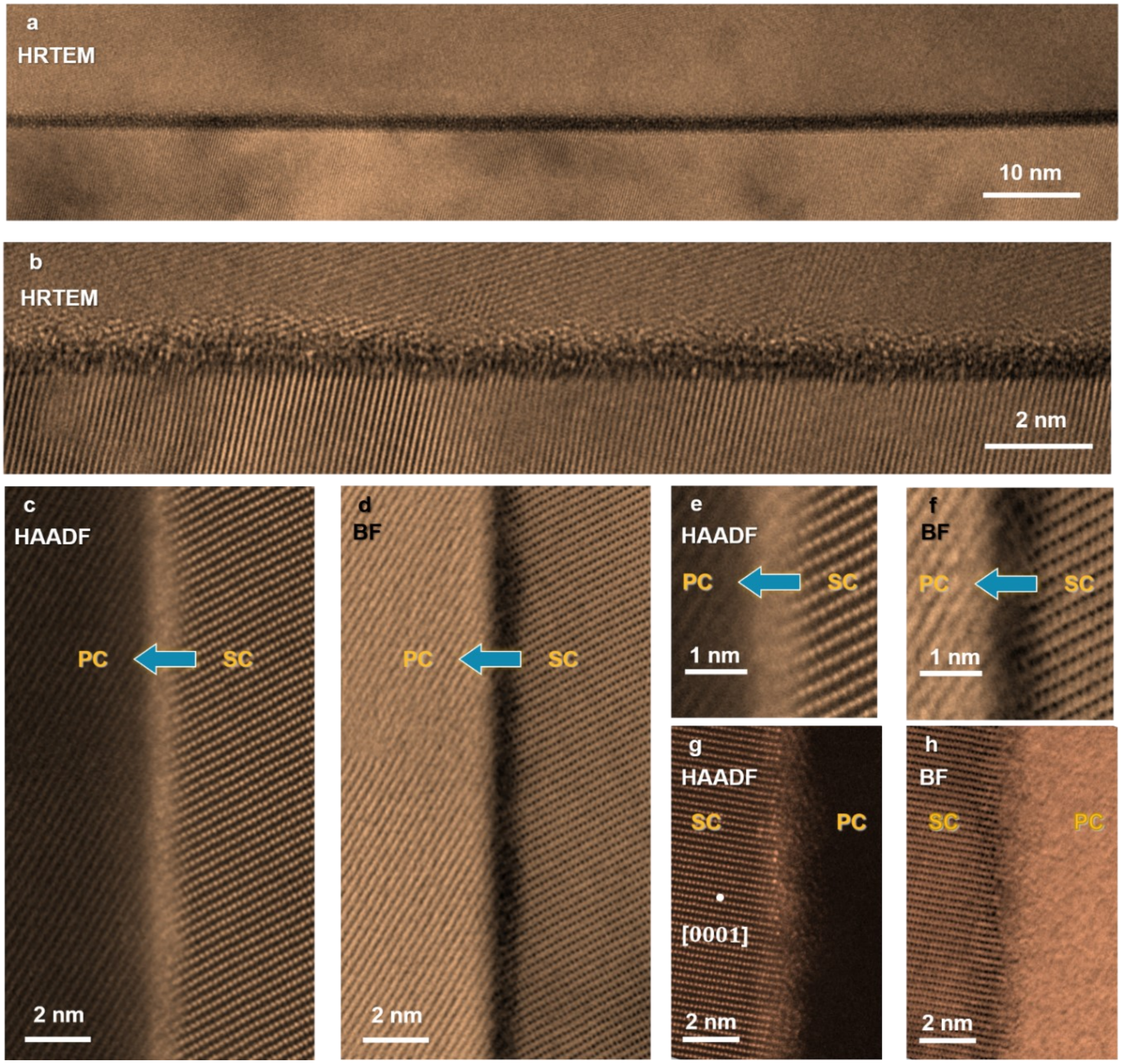

Supplementary Fig. 9. High-resolution transmission electron microscopy (HRTEM) and AC STEM images of the (slow-moving) disordered GBs in Bi_2O_3-doped ZnO specimens annealed at 880°C with no electric field. a, **b**, HRTEM images and **c-h**, AC STEM HAADF and BF images of the characteristic "amorphous-like" intergranular films (IGFs) (*a.k.a.* "disordered" GBs) in Bi_2O_3-doped ZnO specimens quenched from 880 °C after annealing for 4 hours with no electric field. Panels (e) and (f) are an enlarged view of the GB shown in Fig. 1a, b and all others are additional images to show the ubiquitous formation of such disordered GBs in Bi_2O_3-doped ZnO. Such nanoscale amorphous-like IGFs have been observed to form at all general GBs in Bi_2O_3-saturated ZnO at thermodynamic equilibria both above and below the bulk eutectic temperature (740 °C) in prior studies[67,73,74].

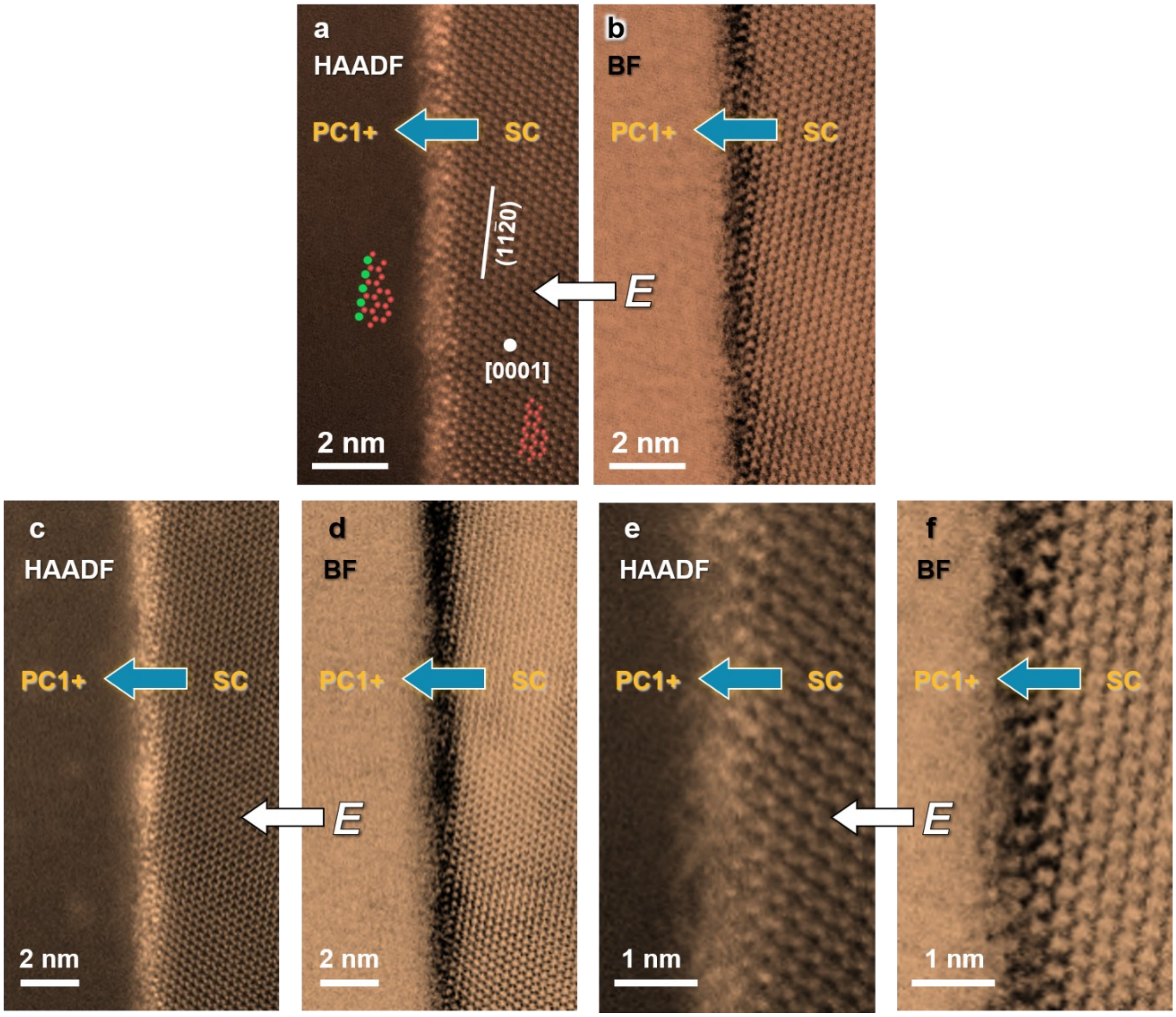

Supplementary Fig. 10. HAADF and BF STEM images of the (slow-moving) disordered GBs in the oxidized PC1+ region. a, **b**, An enlarged view of the GB shown in Fig. 1e, f. **c-f**, Additional AC-STEM images showing the ubiquitous observations of disordered GBs of similar character in the oxidized PC1+ region of the Bi_2O_3-doped ZnO sandwich specimen annealed with constant J = 6.4 mA/mm^2.

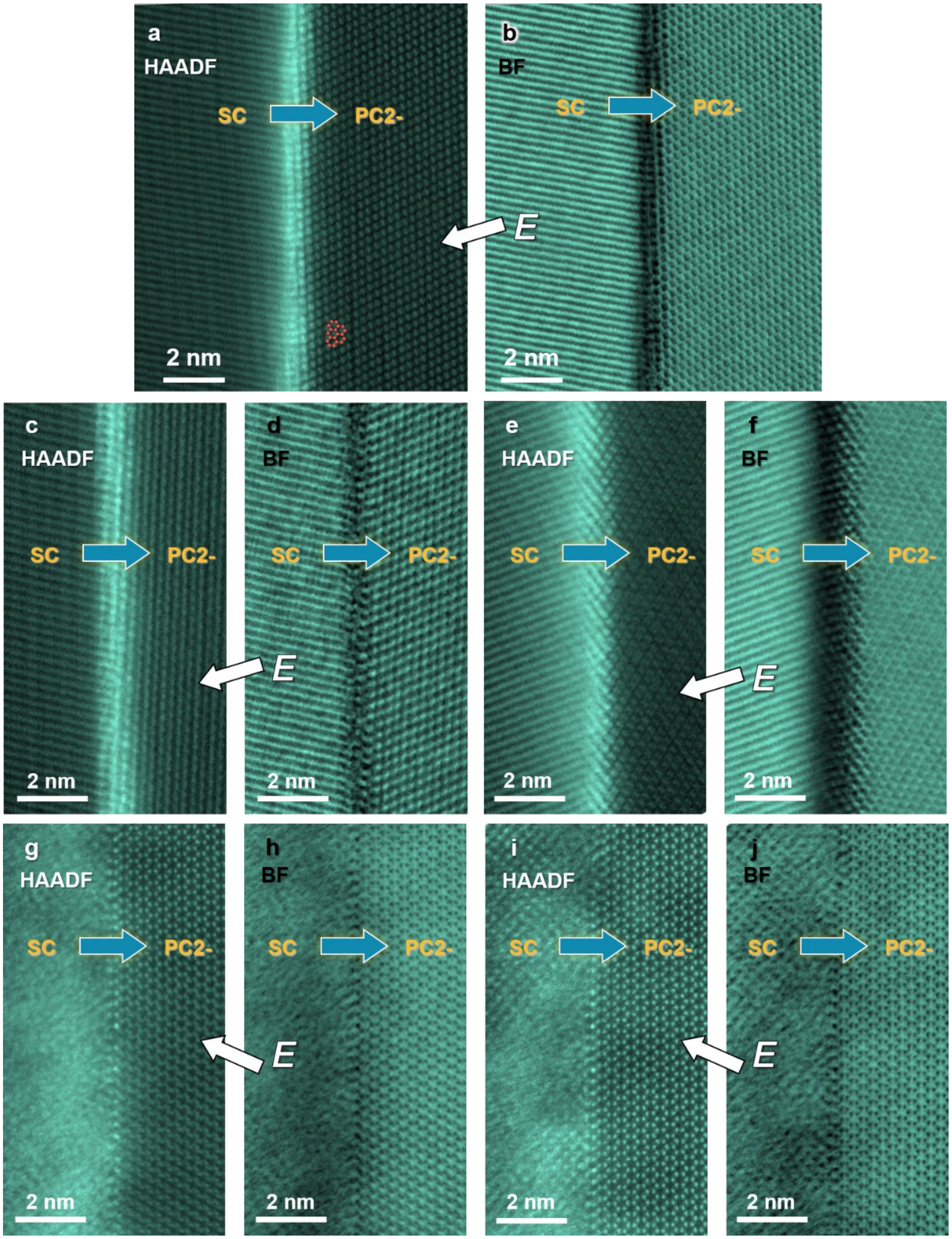

Supplementary Fig. 11. HAADF and BF STEM images of the (fast-moving) ordered GBs in the reduced PC2- region. STEM images of representative (**a-f**) bilayer- or multilayer-like and (**g-j**) monolayer-like ordered GB complexions. This specimen was annealed with constant 6.4 mA/mm^2. Panels (a) and (b) are a larger view of the GB shown in Fig. 1g, h and all others are more AC-STEM images showing the ubiquitous observations of ordered GBs in the reduced PC2- region of the ZnO-Bi_2O_3 sandwich specimen. The fast-moving ordered GB did not maintain the initial orientation; the images are rotated so that the GBs are vertical, where the field directions are indicated by the arrows.

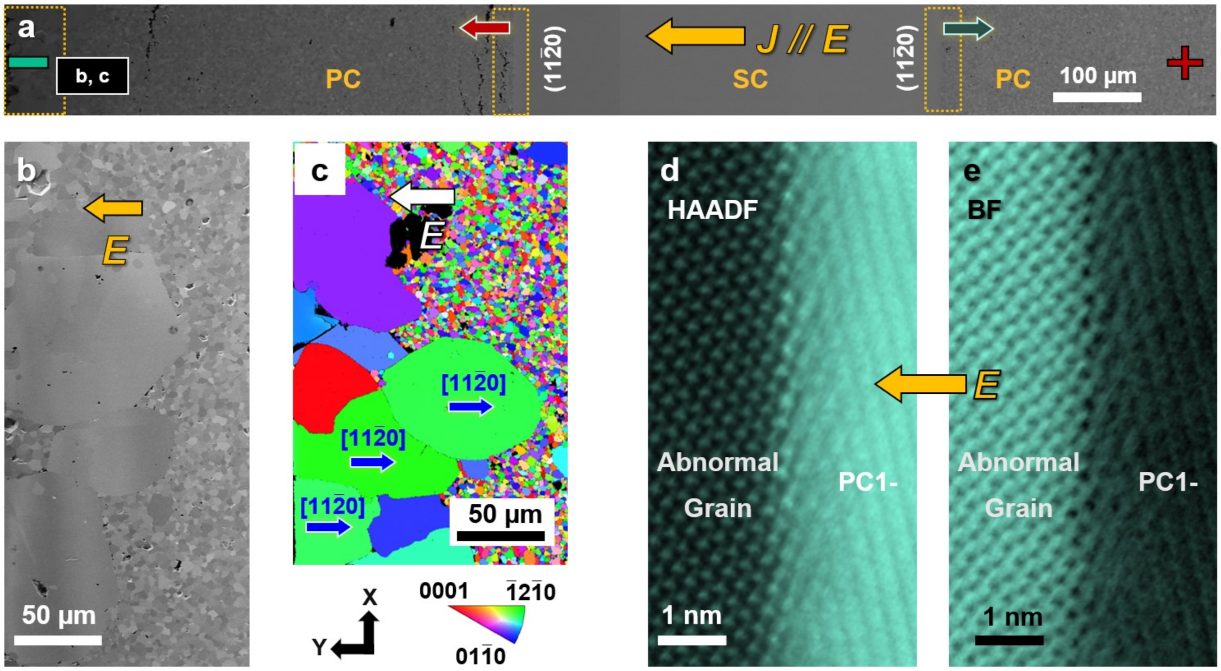

Supplementary Fig. 12. Microstructure and crystallography of the abnormal grains near the cathode in the reduced PC1- region in the sandwich specimen and EBSD map of an additional polycrystalline specimen, both annealed with applied electric currents. a, **b**, Cross-sectional SEM of the abnormal grains near the cathode of this specimen. **c,** Y-direction inverse pole figure (Y-IPF) in EBSD micrographs of the abnormal grains. **d**, **e**, AC HAADF and BF STEM images of a fast-moving, ordered GB between an abnormal grain and an abutting smaller (normal) grain. This specimen was annealed with constant $J = 6.4$ mA/mm^2.

EDS Confirmation of Bi Segregation: The bright contrasts at GBs in the HAADF images are due to heavy Bi adsorbates (because of the *Z* contrast). Bi segregation has also been directly verified by energy dispersive X-ray spectroscopy (EDS) analysis (Supplementary Fig. 13). Note that this GB was intentionally tilted slightly to reduce beam damages (i.e., the GB is not exactly edge-on, so that the Bi segregation region looks wider in the Bi elemental map).

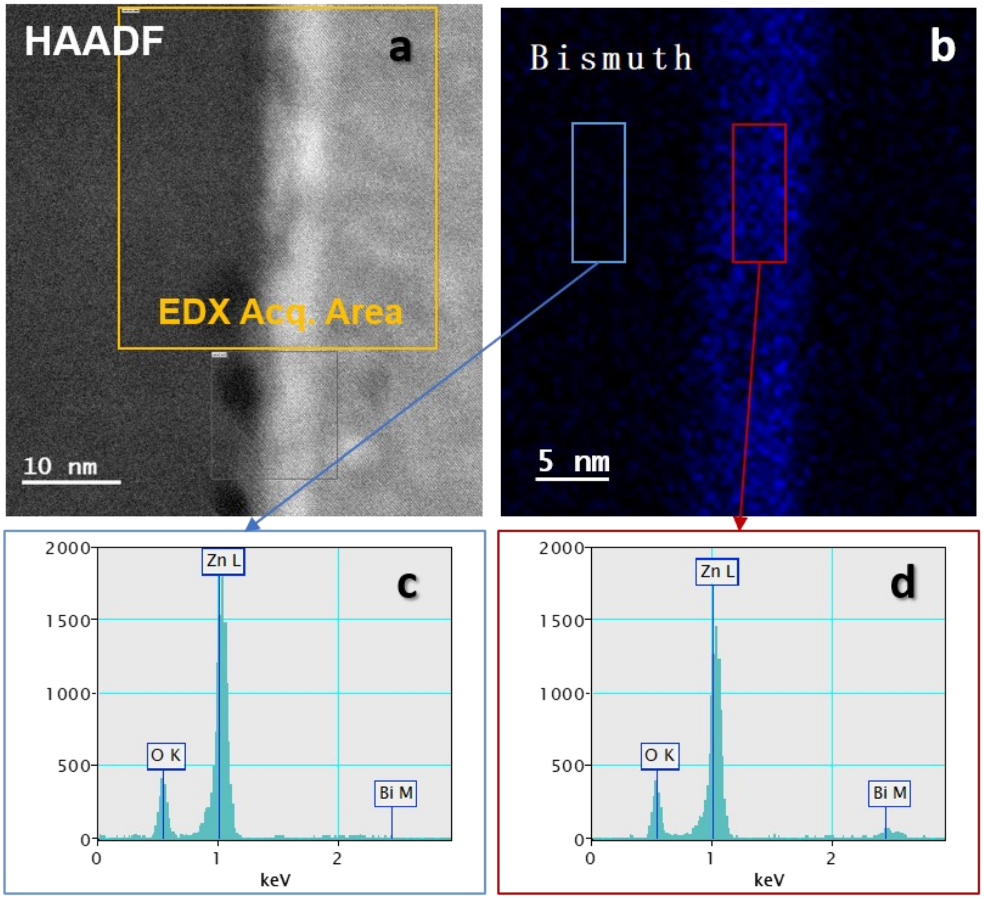

Supplementary Fig. 13. Energy dispersive X-ray spectroscopy (EDS) confirming the GB segregation of Bi. a STEM HAADF image, **b**, EDS Bi elemental map, and EDS spectra of two selected areas **c,** inside the ZnO grain and **d,** at the Bi-enriched GB, respectively. This GB was in the reduced PC1- region in the Bi_2O_3-doped ZnO specimen quenched after annealing for 4 hours with the constant current density of 6.4 mA/mm^2. Noting that this GB was intentionally tilted slightly to reduce beam damages (*i.e.*, the GB is not exactly edge-on, so that the Bi segregation region looks wider in the Bi elemental map). It is known that Bi_2O_3 adsorbates can be damaged (and presumably reduced) rapidly by the irradiation of an intense electron beam in high vacuum in a TEM (as shown for analogous surface phase in Bi_2O_3-doped ZnO in a prior study [83]).

Supplementary Note 6:

DFT optimized GB structures and comparisons with experiments

The procedure for constructing GB models and the details of the density functional theory (DFT) calculations can be found in Methods. A few key points are summarized as follows. A low-symmetry GB model (containing 240 atoms) was selected to represent general GBs, where one terminal GB plane was set to be $(11\bar{2}0)$ to best mimic the SC/PC interface in our experiment within the size limit of the DFT calculations. To dope stoichiometric GBs, we added one oxygen atom for every two Bi dopants (normally forming two Bi^{3+} cations to replace two Zn^{2+} cations) to keep the charge neutrality and fully relaxed the GB structures. In most calculations, the amount of GB excess of Bi was selected to match the experimentally measured average value from prior studies [67,74]. In each case, the GB free volume was allowed to be relaxed to achieve an equilibrium.

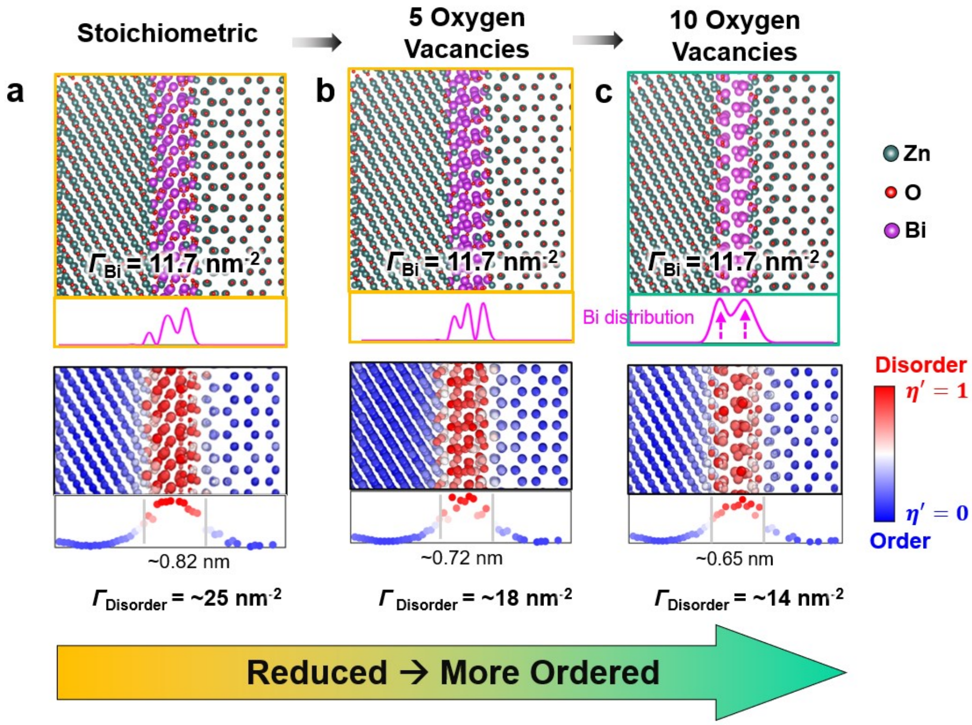

Supplementary Fig. 14. Variation of GB structures with increasing levels of reduction predicted by DFT. a, DFT-relaxed stoichiometric ZnO-Bi_2O_3 GB structure with a Γ_{Bi} of 11.7 Bi atoms nm^{-2} (equivalent to about one monolayer of Bi coverage, which is about the averaged value from prior STEM EDS measurements [55]). Note that one oxygen atom is added into the GB region after doping every two Bi atoms (to replace two Zn atoms) to keep the stoichiometry. **a**, The DFT-relaxed stoichiometric GB (mimic the oxidized condition in experiments). The DFT-relaxed reduced GB structures by (**b**) removing ~5.8 oxygen atoms per nm^2 (about half monolayer) and (**c**) ~11.7 oxygen atoms per nm^2 (about one monolayer), respectively. The computed Bi distribution and disorder profiles indicate that oxygen reduction leads to the formation of more ordered GB structures.

To simulate reduced GBs, we removed a controlled number of oxygen atoms and fully relaxed the interfacial structure again. A series of DFT optimized interfacial structures of stoichiometric

and reduced GBs are shown in Supplementary Fig. 14, which reveal that reduction can make the GB more ordered. To better quantify the GB disorder, we calculated a dimensionless disorder parameter (η' = 1 for an atom in a liquid and η' = 0 for an atom in a perfect crystal; $\eta' = 1 - \eta$, where η is an order parameter; see Methods for the definition and quantification method).

Noting that the DFT-relaxed stoichiometric GB represent the GB observed in the oxidized conditions in experiments (since we expect that no excess oxygen at GBs in experiments).

Then, we computed the GB excess disorder (Γ_{Disorder}) by integrating the $\eta'(x)$ profile for a GB located at x = 0 based on the methods used in prior studies [75-78].

As shown in Supplementary Fig. 14, the GB excess disorder decreased from Γ_{Disorder} = ~25 nm^{-2} for the stoichiometric amorphous-like GB to Γ_{Disorder} = ~18 nm^{-2} after removing about half monolayer of oxygen atoms (*i.e.*, ~5.8 oxygen atoms per nm^2) and Γ_{Disorder} = ~14 nm^{-2} after removing about one monolayer of oxygen atoms (*i.e.*, ~11.7 oxygen atoms per nm^2), respectively.

Specifically, the reduced GB shown in Supplementary Fig. 14c (after removing about one monolayer of oxygen atoms) exhibit a characteristic interfacial structure of bilayer-like Bi adsorption resembling that observed at the SC/PC2- interface by AC STEM in Fig. 5b in the main article. Thus, we show this ordered (bilayer-like) reduced GB along with the amorphous-like GB in Fig. 5a and critically compare them with STEM HAADF images.

Specifically, Fig. 5a-f in the main article compares experimentally observed and DFT simulated stoichiometric (disordered) *vs.* reduced (ordered) GB structures:

- Expanded views of STEM HAADF images for a stoichiometric and disordered GB *vs.* a reduced and ordered (bilayer-like) GB are shown in Fig. 5a *vs.* 4b, where the averaged intensity and line-by-line FFT patterns are also plotted to illustrate the layering and periodic orders. Here, DFT-relaxed stoichiometric GB represent the GB observed in the oxidized conditions in experiments (as discussed above).
- The STEM images are compared with DFT-optimized structures of the stoichiometric *vs.* reduced GBs shown in Fig. 5c *vs.* 4d, where the Bi concentration profiles projected along the *x* direction are plotted beneath.
- The calculated disorder parameters for all atoms for DFT-optimized stoichiometric *vs.* reduced GB structures are shown in Fig. 5e *vs.* 4f. The disorder parameter profiles $\eta'(x)$ projected along the *x* direction are plotted above.

Overall, DFT calculations agree well with the experimental observations:

- On the one hand, the stoichiometric GB is disordered, as shown in the STEM image and FFT analysis in Fig. 5a, with a computed Γ_{Disorder} of ~25 nm^{-2}. The width of the disordered layer (IGF) was measured to be ~0.9 nm from the STEM HAADF image shown in Fig. 5a (and ~0.7-0.9 nm for different IGFs observed in this study) and ~0.8 nm from the DFT simulated disorder profile (Fig. 5e), which agree with each other.
- On the other hand, the reduced GB is more ordered with a bilayer-like structure shown in

both the STEM image (Fig. 5b) and the Bi adsorption profile obtained by the DFT optimization (Fig. 5c), with a reduced computed $\Gamma_{Disorder}$ of ~14 nm^{-2}. Both the STEM HAADF image and DFT calculations show that the interlayer distance (within the bilayer-like interfacial structure) is ~0.3 nm.

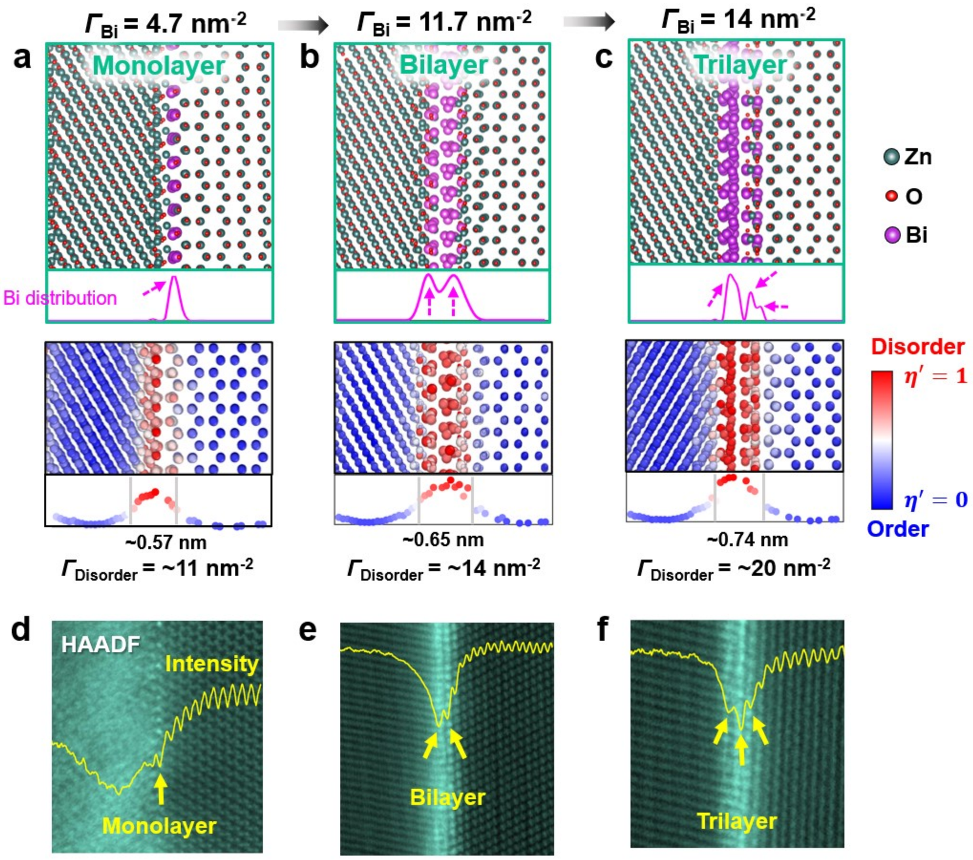

Supplementary Fig. 15. A series of ordered complexions (Bi adsorption structures) formed at electrochemically reduced GBs. a-c, DFT-optimized reduced GB structures with increasing Bi adsorption (Γ_{Bi}), showing the formation of monolayer-, bilayer-, and trilayer-like complexions. **d-f**, STEM HAADF images of ordered GBs observed in the reduced regions of our specimen that resemble these layered adsorption structures predicted from DFT (albeit different GB characters). It is noted that experimental observations were made on GBs randomly-selected from the reduced region, which cannot be modeled accurately due the lacking of full details of the GB crystallographic characters and the limitations of the maximum size for effective DFT calculations.

In addition to GB bilayer-like structure, DFT calculations show that reduced ZnO GBs can also exhibit a range of ordered segregation structures, from monolayer to trilayer with increasing levels of GB disorder and interfacial width Supplementary Fig. 15). Interestingly, similar interfacial structures have also been observed in randomly selected general GBs in the electrochemically reduced regions in STEM HAADF images (Supplementary Fig. 15d-f) in this study. More critical assessments are not warranted here because we could not determine the exact crystallographic characters of randomly-selected general GBs from experiments in most cases to build cells to allow DFT calculations (which are also limited for most general GBs because realistic

models often contain too many atoms). Nevertheless, these experimental discoveries, along with DFT modeling, demonstrate the generality of forming ordered GB structures in the reduced regions of Bi_2O_3-doped ZnO.

In summary, DFT calculations have confirmed that the reduction can induce a GB disorder-to-order transition in Bi_2O_3-doped ZnO, and the predictions are consistent with experiments.

Supplementary Note 7:

DFT calculations of GB energies and a predicted GB disorder-order transition

To further verify and model the reduction-induced GB disorder-order transition, we calculated the GB energy as a function of chemical potentials for both stoichiometric and reduced GBs. The GB energy (γ_{GB}) can be determined from DFT calculations using the following equation:

$$\gamma_{\mathrm{GB}} = \frac{E_{\mathrm{Total}}^{GB\ Bi@ZnO} - E_{\mathrm{Grain1}}^{ZnO} - E_{\mathrm{Grain2}}^{ZnO} - n_{\mathrm{O}}\mu_{\mathrm{O}} + n_{\mathrm{Bi/Zn}}\mu_{\mathrm{Zn}} - n_{\mathrm{Bi/Zn}}\mu_{\mathrm{Bi}}}{A}, \quad (4)$$

where $E_{\mathrm{Total}}^{GB\ Bi@ZnO}$ is the total energy of the Bi-doped ZnO DFT calculation cell with the GB (after the full relaxation), $E_{\mathrm{Grain1(2)}}^{ZnO}$ is the reference energy of Grain 1 (or 2), $n_{\mathrm{Bi/Zn}}$ is the number of Zn atoms substituted by Bi atoms, n_{O} is the number of excess oxygen atoms (*i.e.*, number of the oxygen atoms added at the GB to compensate the charge of aliovalent Bi doping minus the number of the oxygen atoms removed for creating reduction), μ_i (i = O, Zn, or Bi) is the chemical potential of O, Zn, or Bi, and *A* is the cross-sectional area of the GB. The oxygen chemical potential difference $\Delta\mu_{\mathrm{O}}$ can be defined as:

$$\Delta\mu_{\mathrm{O}} = \mu_{\mathrm{O}} - \frac{1}{2}E_{\mathrm{O_2}}, \quad (5)$$

where $E_{\mathrm{O_2}}$ is the energy of the oxygen gas molecule with a correction [82]. The boundaries of $\Delta\mu_{\mathrm{O}}$ are given by:

$$\Delta\mu_{\mathrm{O}} < 0, \quad (6)$$

and

$$\Delta\mu_{\mathrm{O}} + \Delta\mu_{\mathrm{Zn}} = \Delta H_f(\mathrm{ZnO}), \quad (7)$$

where $\Delta H_f(\mathrm{ZnO})$ is the formation enthalpy of ZnO. Similarly, we define the Bi chemical potential difference as:

$$\Delta\mu_{\mathrm{Bi}} = \mu_{\mathrm{Bi}} - E_{\mathrm{Bi}}, \quad (8)$$

where E_{Bi} is the DFT energy of the Bi element (in its stable solid form). The upper and lower boundaries of $\Delta\mu_{\mathrm{Bi}}$ can be specified by a similar method.

Using equations (5-8), we calculated GB energies for both stoichiometric and reduced GBs. In the Supplementary Fig. 16, we plotted DFT-calculated GB energies γ_{GB} of stoichiometric/disordered and reduced/ordered GBs as a function of chemical potential difference of oxygen ($\Delta\mu_{\mathrm{O}}$) and bismuth ($\Delta\mu_{\mathrm{Bi}}$), where the intersection line between the $\gamma_{\mathrm{GB}}^{\mathrm{Reduced}}$ and $\gamma_{\mathrm{GB}}^{\mathrm{Stoichiometric}}$ planes define a GB transition.

Subsequently, we define the GB energy difference, $\Delta\gamma_{\mathrm{GB}}$, as:

$$\Delta\gamma_{\mathrm{GB}} = \gamma_{\mathrm{GB}}^{\mathrm{Reduced}} - \gamma_{\mathrm{GB}}^{\mathrm{Stoichiometric}}, \quad (9)$$

where $\gamma_{\mathrm{GB}}^{\mathrm{Stoichiometric}}$ and $\gamma_{\mathrm{GB}}^{\mathrm{Reduced}}$, respectively, are the GB energies for stoichiometric (disordered) and reduced (ordered) GBs, respectively, calculated from DFT. Here, $\Delta\gamma_{\mathrm{GB}} = 0$ defines a transition.

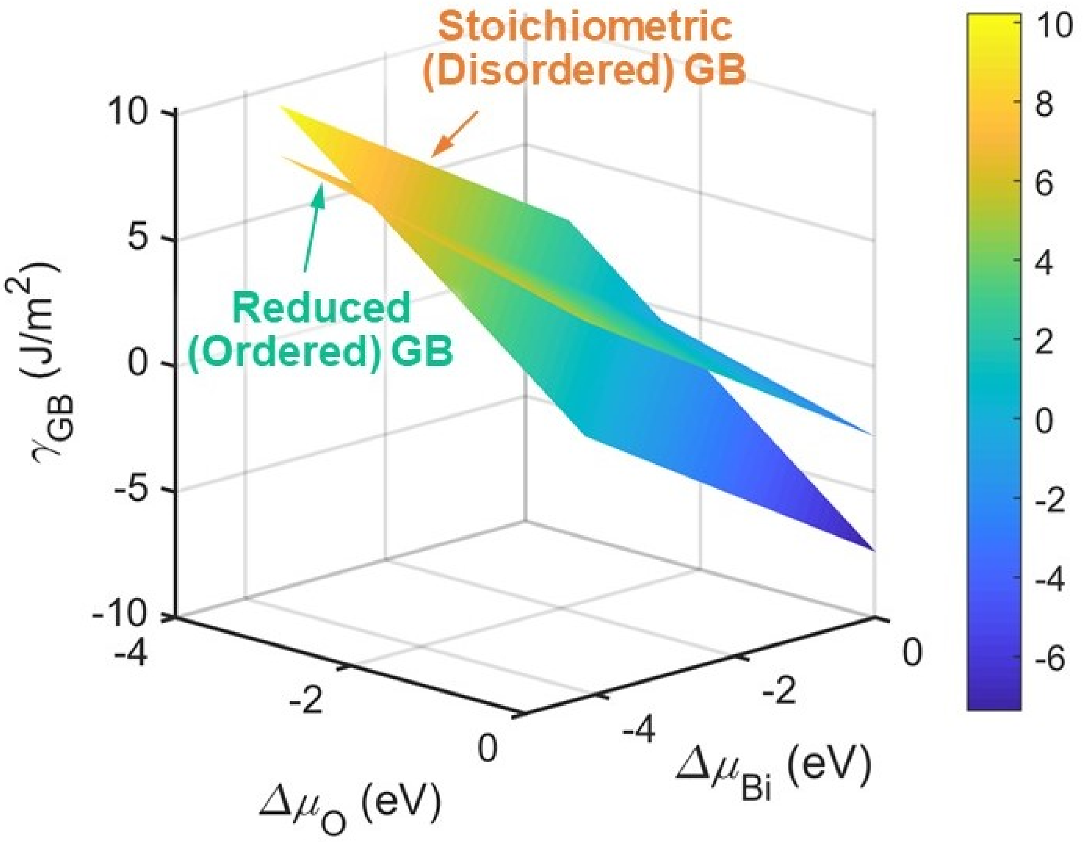

Supplementary Fig. 16. DFT calculated GB energies to predict the transition between the stoichiometric and reduced GBs. DFT calculated GB energies γ_{GB} of stoichiometric (disordered) and reduced (ordered) GBs as a function of chemical potential difference of oxygen ($\Delta\mu_{\mathrm{O}}$) and bismuth ($\Delta\mu_{\mathrm{Bi}}$). Note that this calculation adopted same GB excess of Bi adsorption for both GBs, so that the difference in the GB energies $\Delta\gamma_{\mathrm{GB}}$ (= $\gamma_{\mathrm{GB}}^{\mathrm{Reduced}} - \gamma_{\mathrm{GB}}^{\mathrm{Stoichiometric}}$) is independent of $\Delta\mu_{\mathrm{Bi}}$. Consequently, a simplified plot of $\Delta\gamma_{\mathrm{GB}}$ as a function of $\Delta\mu_{\mathrm{O}}$ can be plotted in Fig. 6a.

Since both stoichiometric and reduced GBs have the same amount of GB excess of Bi, the difference in the GB energies $\Delta\gamma_{\mathrm{GB}}$ is in fact independent of $\Delta\mu_{\mathrm{Bi}}$. Thus, we can simplify Supplementary Fig. 16. Consequently, Fig. 6a shows a simplified plot of $\Delta\gamma_{\mathrm{GB}}$ as a function of $\Delta\mu_{\mathrm{O}}$ that represents this GB transition from a stoichiometric (disordered) GB to a reduced (ordered) GB.

Supplementary Note 8:
A generalizable thermodynamic model supported by DFT calculations

Here, we further discuss the physical origin of the reduction-induced GB disorder-to-order transition (*a.k.a.* why reduction can induce GB ordering) via a generalizable phenomenological thermodynamic model supported by DFT calculations.

Tang, Carter, and Cannon proposed a diffuse-interface model for a GB disorder-order transition, where the excess grand potential for a two-component GB is given by [79]:

$$\sigma^x = \int_{-\infty}^{+\infty} \left[\Delta f(\eta, c) + \frac{\kappa_\eta^2}{2} \cdot \left(\frac{d\eta}{dx} \right)^2 + \frac{\kappa_c^2}{2} \cdot \left(\frac{dc}{dx} \right)^2 + s \cdot g(\eta) \cdot \left| \frac{d\theta}{dx} \right| \right] dx \, . \tag{10}$$

Here, the profiles of concentration $c(x)$, crystallinity $\eta(x)$ ($= 1 - \eta'(x)$, where η' is the disorder parameter), and crystallographic orientation $\theta(x)$, are functions of x, the spatial parameter perpendicular to a GB located at $x = 0$; $\Delta f(c, \eta)$ is the homogenous (bulk) free energy density referred to the equilibrium bulk phases. Gradient energy coefficients, κ_η, κ_c, and s, are model parameters (materials constants). In equation (10), $g(\eta)$ is a function characterizing the coupling between $|d\theta / dx|$ and η, and we can assume that $g(\eta) = \eta^2$ (consistent with the Read-Shockley model) for simplicity [80]; high order terms can be included but this simple form can capture the basic underlying physics of the order-disorder transitions [79,81] so that it is adopted here. The equilibrium $c(x)$, $\eta(x)$, and $\theta(x)$ profiles should minimize the excess grand potential (*a.k.a.* interfacial energy) in equation (10).

Moreover, it was proven that $\theta(x)$ should take a step function [79,80]. Subsequently, minimization of the excess grand potential (interfacial energy) in equation (10) leads to [79]:

$$\sigma^x = s \cdot \Delta\theta \cdot \eta_{\mathrm{GB}}^2 + \Delta F(\eta_{\mathrm{GB}}), \tag{11}$$

where η_{GB} is the order parameter at $x = 0$, $\Delta\theta$ is the GB misorientation, and

$$\Delta F(\eta_{\mathrm{GB}}) \equiv \min \left\{ 2 \cdot \int_{\eta_{\mathrm{GB}}}^{1} \sqrt{2\Delta f(\eta, c) \cdot \left[\kappa_\eta^2 + \kappa_c^2 \cdot \left(\frac{dc}{d\eta} \right)^2 \right]} d\eta \right\}. \tag{12}$$

In equation (11), the first term $s \cdot \Delta\theta \cdot \eta_{GB}^2$ represents an energetic penalty to have a GB misorientation $\Delta\theta$, where GB disordering (or a smaller η_{GB}) lowers this energetic penalty. The second term $\Delta F(\eta_{\mathrm{GB}})$ represents the total increased free energy due to GB disordering and associated compositional variation (*i.e.*, GB segregation) and gradient energy penalties (from the second and third terms in the integral in equation (10)) in a diffuse interface. From the definition in equation (12), we know:

$$\Delta F\left(\eta_{\text{GB}}=1\right)=0 \tag{13}$$

and

$$\Delta F\left(\eta_{\text{GB}}<1\right)>0\,. \tag{14}$$

In equation (12), the value of the integral also depends on the function $c(\eta)$ and c_{GB}, the composition at the GB. In a prior work [79], Tang, Carter, and Cannon proved (via a slightly different approach) that the following relations must be held for an equilibrium GB in a binary alloy to specify $\eta_{\text{GB}}^{\text{Equilibrium}}$ and $c_{\text{GB}}^{\text{Equilibrium}}$ at a thermodynamic equilibrium:

$$s\cdot\Delta\theta\cdot\eta_{\text{GB}}^{\text{Equilibrium}}=\sqrt{2\kappa_{\eta}^{2}\cdot\Delta f\left(\eta_{\text{GB}}^{\text{Equilibrium}},c_{\text{GB}}^{\text{Equilibrium}}\right)}\,. \tag{15}$$

The two boundary conditions for the function $c(\eta)$ are:

$$c(\eta=1)=c_{\infty}\,, \tag{16}$$

where c_{∞} is the composition inside the bulk (grain), and

$$\left.\frac{dc}{d\eta}\right|_{\eta_{\text{GB}}^{\text{Equilibrium}}}=0\,. \tag{17}$$

Subsequently, they developed a 3D graphical construction method to solve $\eta_{\text{GB}}^{\text{Equilibrium}}$ and $c_{\text{GB}}^{\text{Equilibrium}}$ simultaneously based on equations (15-17) [79].

Here, we simplify that approach to allow 2D graphical construction (to help illustrate the key underlying physical picture intuitively) by first adopting the $c(\eta)$ profile and $c_{\text{GB}}(\eta_{\text{GB}})$ that minimize $\Delta F\left(\eta_{\text{GB}}\right)$ for a given η_{GB} in equation (12). Then, we can solve the equilibrium level of the GB order (or GB disorder $\eta'_{\text{GB}}\equiv 1-\eta_{\text{GB}}$) by finding $\eta_{\text{GB}}^{\text{Equilibrium}}$ (only one variable) to minimize the excess grand potential (interfacial energy) in equation (11) via differentiation:

$$2s\cdot\Delta\theta\cdot\eta_{\text{GB}}^{\text{Equilibrium}}=-\left.\frac{d(\Delta F)}{d\eta_{\text{GB}}}\right|_{\eta_{\text{GB}}^{\text{Equilibrium}}}\,. \tag{18}$$

By comparing equations (15) and (18), we know:

$$-\left.\frac{d(\Delta F)}{d\eta_{\text{GB}}}\right|_{\eta_{\text{GB}}^{\text{Equilibrium}}}=2\sqrt{2\kappa_{\eta}^{2}\cdot\Delta f\left(\eta_{\text{GB}}^{\text{Equilibrium}},c_{\text{GB}}^{\text{Equilibrium}}\right)}\,, \tag{19}$$

where $c_{\text{GB}}^{\text{Equilibrium}}$ is a function of $\eta_{\text{GB}}^{\text{Equilibrium}}$ (that we can solve numerically via the minimization in equation (12)). This equation is similar to Tang, Carter, and Cannon's prior analysis of the unary GB [80], except that a more complex form of $\Delta F\left(\eta_{\text{GB}}\right)$ for a binary GB without an analytical

expression. But $\Delta F(\eta_{\mathrm{GB}})$ is well defined by the minimization in equation (12). Noting that equations (12) and (18) are mathematically equivalent to the equations (15-17) from the Tang-Carter-Cannon model [79]. Here, we rearrange the order of energy minimization to allow us to draw 2D sketches for illustrating disorder-order transitions in a binary alloy without losing the rigorousness.

In the current case, we do not have all parameters to evaluate the Bi_2O_3-doped ZnO system quantitatively. Thus, we first schematically show that oxygen reduction can induce a GB disorder-to-order transition by decreasing the coupling coefficient *s*, (if it can be decreased) and subsequently justify by DFT calculations that the parameter *s* can be decreased by ~2.4× in the reduced GB.

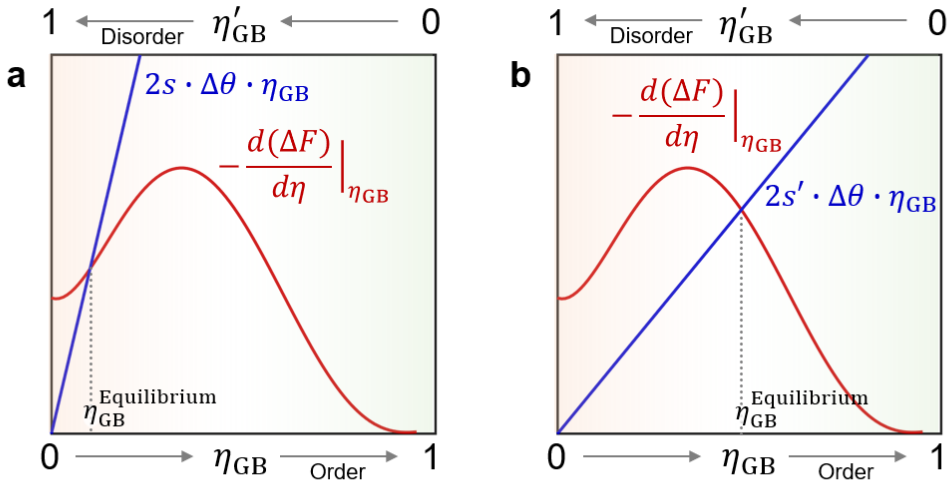

Supplementary Fig. 17. Schematic illustration of the GB disorder-to-order transition using a generalizable thermodynamic model following Tang, Carter, and Cannon[79,81]. a, Schematic illustration of a graphical construction method to solve the equation (2) in the main article or equation (18) here. The intersection of the red and blue lines indicates an equilibrium order parameter $\eta_{\mathrm{GB}}^{\mathrm{Equilbrium}}$. **b**, Decreasing the GB misorientation-disordering coupling parameter (*i.e.*, changing $s \rightarrow$ a smaller s') can change the GB into an ordered GB state (with greater $\eta_{\mathrm{GB}}^{\mathrm{Equilbrium}}$). Since DFT calculations showed that the parameter *s* for the reduced GB is substantially smaller than that for the stoichiometric GB (*i.e.*, decreasing *s* by ~2.4× based on our DFT estimations), oxygen reduction can cause such a GB disorder-to-order transition. Thus, it explains the origin of the observed reduction-induced GB ordering in our experiments.

Equation (18) allows a 2D graphical construction to solve $\eta_{\mathrm{GB}}^{\mathrm{Equilibrium}}$ by plotting the both sides of the equation and finding the intersections. Equation (19) suggests that the right side of equation (18) (*i.e.*, the red curves in Supplementary Fig. 17) would likely to have two minima with a barrier in between because the function $\Delta f(c, \eta)$ has two minima for the liquid ($\eta = 0$) and solid ($\eta = 1$) phases. The Supplementary Fig. 17 schematically illustrates the GB disorder-order transition. The intersection of the red and blue lines in Supplementary Fig. 17a defines an equilibrium order parameter $\eta_{\mathrm{GB}}^{\mathrm{Equilbrium}}$ for a more disordered GB state. The Supplementary Fig. 17b shows that

decreasing s can change the GB into a more ordered state (with a greater $\eta_{\mathrm{GB}}^{\mathrm{Equilbrium}}$).

To support the above-proposed origin of reduction-induced GB order-to-disorder transition quantitatively, we used DFT calculations of the GB structures before and after relaxations (Supplementary Table 1) to estimate $s \cdot \Delta\theta$ for both the stoichiometric GB and the reduced GB. Before the DFT relaxation, we have:

$$\sigma^{x}(\eta_{\mathrm{GB}}^{(0)}) = s \cdot \Delta\theta \cdot \left(\eta_{\mathrm{GB}}^{(0)}\right)^{2} + \Delta F\left(\eta_{\mathrm{GB}}^{(0)}\right), \tag{20}$$

where $\eta_{\mathrm{GB}}^{(0)}$ is the initial order parameter at center of GB (x = 0) before the DFT relaxation. After the relaxation, the equilibrium GB is obtained, for which we have:

$$\sigma^{x}(\eta_{\mathrm{GB}}^{\mathrm{Equilibrium}}) = s \cdot \Delta\theta \cdot \left(\eta_{\mathrm{GB}}^{\mathrm{Equilibrium}}\right)^{2} + \Delta F\left(\eta_{\mathrm{GB}}^{\mathrm{Equilibrium}}\right), \tag{21}$$

We can use the DFT calculations to estimate $\Delta\sigma_{\mathrm{DFT}}^{x} \equiv \sigma^{x}(\eta_{\mathrm{GB}}^{(0)}) - \sigma^{x}(\eta_{\mathrm{GB}}^{\mathrm{Equilibrium}})$. Subsequently, combining equations (20) and (21) produces:

$$\Delta\sigma_{\mathrm{DFT}}^{x} = s \cdot \Delta\theta \cdot \left[\left(\eta_{\mathrm{GB}}^{(0)}\right)^{2} - \left(\eta_{\mathrm{GB}}^{\mathrm{Equilibrium}}\right)^{2}\right] + \left[\Delta F\left(\eta_{\mathrm{GB}}^{(0)}\right) - \Delta F\left(\eta_{\mathrm{GB}}^{\mathrm{Equilibrium}}\right)\right], \tag{22}$$

or:

$$s \cdot \Delta\theta \cdot \left[\left(\eta_{\mathrm{GB}}^{(0)}\right)^{2} - \left(\eta_{\mathrm{GB}}^{\mathrm{Equilibrium}}\right)^{2}\right] = \Delta\sigma_{\mathrm{DFT}}^{x} + \left[\Delta F\left(\eta_{\mathrm{GB}}^{\mathrm{Equilibrium}}\right) - \Delta F\left(\eta_{\mathrm{GB}}^{(0)}\right)\right]. \tag{23}$$

Noting that the first term in equation (22) dominates the relaxation, so we know:

$$s \cdot \Delta\theta \cdot \left[\left(\eta_{\mathrm{GB}}^{(0)}\right)^{2} - \left(\eta_{\mathrm{GB}}^{\mathrm{Equilibrium}}\right)^{2}\right] > \left[\Delta F\left(\eta_{\mathrm{GB}}^{\mathrm{Equilibrium}}\right) - \Delta F\left(\eta_{\mathrm{GB}}^{(0)}\right)\right] > 0. \tag{24}$$

Thus, we can use a linear Taylor expansion to estimate the second term (that is smaller in its absolute value in comparison with the first term) in equation (22) to the first order of approximation, as:

$$\left[\Delta F\left(\eta_{\mathrm{GB}}^{(0)}\right) - \Delta F\left(\eta_{\mathrm{GB}}^{\mathrm{Equilibrium}}\right)\right] \approx \left.\frac{d(\Delta F)}{d\eta_{\mathrm{GB}}}\right|_{\eta_{\mathrm{GB}}^{\mathrm{Equilibrium}}} .\left(\eta_{\mathrm{GB}}^{(0)} - \eta_{\mathrm{GB}}^{\mathrm{Equilibrium}}\right). \tag{25}$$

Combining with equation (22), we can obtain:

$$\left[\Delta F\left(\eta_{\mathrm{GB}}^{(0)}\right) - \Delta F\left(\eta_{\mathrm{GB}}^{\mathrm{Equilibrium}}\right)\right] \approx -2s \cdot \Delta\theta \cdot \eta_{\mathrm{GB}}^{\mathrm{Equilibrium}} .\left(\eta_{\mathrm{GB}}^{(0)} - \eta_{\mathrm{GB}}^{\mathrm{Equilibrium}}\right). \tag{26}$$

Plugging the above equation into equation (22), we have:

$$\Delta\sigma_{\mathrm{DFT}}^{x} \approx s \cdot \Delta\theta \cdot \left[\left(\eta_{\mathrm{GB}}^{(0)}\right)^{2} - \left(\eta_{\mathrm{GB}}^{\mathrm{Equilibrium}}\right)^{2}\right] - 2s \cdot \Delta\theta \cdot \eta_{\mathrm{GB}}^{\mathrm{Equilibrium}} .\left(\eta_{\mathrm{GB}}^{(0)} - \eta_{\mathrm{GB}}^{\mathrm{Equilibrium}}\right). \tag{27}$$

Hence, we can estimate the $s \cdot \Delta\theta$ (where $\Delta\theta$ is dimensionless constant) via:

$$s \cdot \Delta\theta \approx \frac{\Delta\sigma_{\mathrm{DFT}}^{x}}{\left(\eta_{\mathrm{GB}}^{(0)} - \eta_{\mathrm{GB}}^{\mathrm{Equilibrium}}\right)^2} \tag{28}$$

Based on the DFT calculations of the stoichiometric and reduced GBs before and after the relaxation, we computed $s \cdot \Delta\theta$ for both stoichiometric and reduced GBs. The key results are shown in Supplementary Table 1. Since $\Delta\theta$ is a constant (the GB misorientation), this calculation suggested that the reduction can decrease the coupling parameter/coefficient s by about 2.4 times. Thus, it quantitively justifies that reduction can induce a GB disorder-to-order transition based on Supplementary Fig. 17.

This generalizable thermodynamic model and DFT-based approach can also be useful for predicting trends for other systems in future studies.

We shall make several additional notes for the sake of completeness and rigor (albeit that they are not the focus of the discussion here). First, we expect the red curves in Supplementary Fig. 17 have two minima and one maximum (based on equation (19)), but we do not have an analytical expression (that has to be obtained numerically based on equation (12)); yet the sketches in Supplementary Fig. 17 can clearly illustrate the physical pictures. Second, if the blue line intersects with the red curve twice, it defines two (stable and metastable) GB states and a possible first-order GB transition [79,81], which is a scientifically interesting interfacial phenomenon but not the focus of the discussion here. Third, the blue curve can be nonlinear beyond the adoption of the simple function of $g(\eta) = \eta^2$ (to represent the Ready-Shockley type behavior). Nonetheless, the current approach and sketches in Supplementary Fig. 17, along with the DFT calculations shown in Supplementary Table 1, can effectively illustrate the physical origin of the reduction-induced GB disorder-to-order transition via decreasing the parameter s.

Supplementary Table 1. DFT calculations of the GB structures before and after the relaxations to evaluate the change of *s* to justify the disorder-order transition. E_{relax} and $E_{unrelax}$ are the energies of ZnO GBs after and before the relaxation. $\Gamma_{\mathrm{Disorder}}$ is the GB excess of disorder (listed here for reference). $\eta_{\mathrm{GB}}^{(0)}$ and $\eta_{\mathrm{GB}}^{\mathrm{Equlibirum}}$ are the order parameter at the center of unrelaxed and relaxed GB (at *x* = 0). The parameter *s* is decreased by ~2.4X in the reduced GB, which can justify the reduction-induced GB disorder-to-order transition.

	**Stoichiometric GB**	**Reduced GB**
E_{relax} (eV/atom)	-4.480	-4.385
$E_{unrelax}$ (eV/atom)	-3.718	-4.060
$\Delta\sigma_{\mathrm{DFT}}^{x}$ (eV/nm^2)	218	42
$\Gamma_{\mathrm{Disorder}}^{\mathrm{Equilibrium}}$ (nm^{-2})	25	14
$\Gamma_{\mathrm{Disorder}}^{(0)}$ (nm^{-2})	14	11
$\eta_{\mathrm{GB}}^{\mathrm{Equilibirum}}$	0.10	0.18
$\eta_{\mathrm{GB}}^{(0)}$	0.35	0.35
$s \cdot \Delta\theta$ (eV/nm^2)	~3.5×10^3	~1.4×10^3

Supplementary Note 9:

Mechanisms of enhanced kinetics in the reduced GB: insights from the DFT calculated differential charge densities and Bader charges

To further explain the underlying mechanisms of the enhanced GB kinetics (diffusivities and mobilities) of the reduced GBs, we calculated differential charge density and Bader charge transfers of the Bi atoms for the stoichiometric GB *vs.* the reduced GB.

First, the DFT-calculated isosurfaces, where yellow regions represent charge accumulation and cyan regions represent charge depletion, indicate stronger charge transfer in the stoichiometric GB than that in the reduced GB (Supplementary Fig. 18a *vs*. 18b). By comparing the 2D averaged differential charge density profile along *z* direction (Supplementary Fig. 18c *vs*. 18d), the large oscillation peaks for the stoichiometric GB verified the stronger charge transfer. Since atoms typically form strong chemical bonding with strong charge transfer, this stronger charge transfer can explain the low GB diffusivities in, and the mobility of, the stoichiometric GBs (because of the stronger bonding or "pinning" effects).

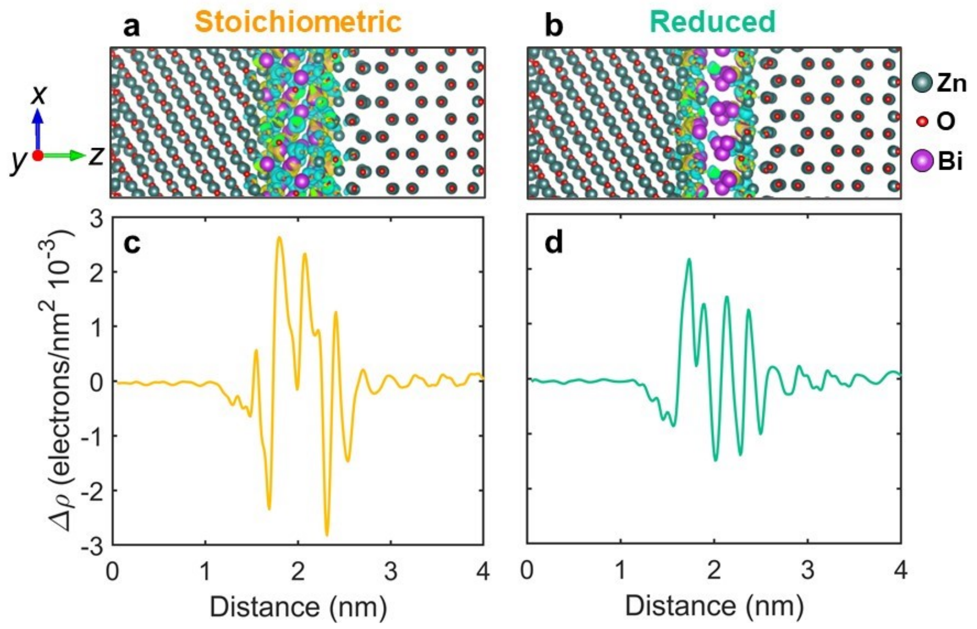

Supplementary Fig. 18. Differential charge densities calculated from DFT to reveal the underlying mechanism of enhanced kinetics in the reduced GB. Isosurfaces of the differential charge densities of (**a**) stoichiometric GB and (**b**) reduced GB. The isovalue is set to 0.01 e/Å^3 for the plots. The yellow and cyan isosurfaces, respectively, represent charge accumulation and depletion, respectively. The differential charge density profiles projected along the *z* direction of (**c**) stoichiometric GB and (**d**) reduced GB, suggesting weaker bonding in the reduced (ordered) GB

Second, the calculated average Bader charges showed that one Bi atom losses ~1.4 *e* in the stoichiometric GB, which is significantly greater than ~0.69 *e* in the reduced GB (Supplementary Table 2). Thus, the aliovalent Bi atoms serve as charged "hot spots" to provide more "pinning"

effects in the stoichiometric GB, thereby decreasing the GB diffusivities and mobility. However, the oxygen reduction may significantly reduce the charged "hot spots" and associated strong bonding and "pinning" effects, thereby enhancing the GB diffusivities in, and the mobility of, the reduced GBs, which subsequently caused the enhanced and abnormal grain growth observed in experiments.

The above thermodynamic model and DFT results can be understood intuitively. On the one hand, the presence of aliovalent Bi^{3+} adsorbates (substituting Zn^{2+} cations with extra O^{2-} for the charge compensation) in the stoichiometric GB will likely lead to interfacial disordering. On the other hand, the reduction will decrease the effective charge on Bi adsorbates (as shown by calculated Bader charges given in Supplementary Table 2) to make interfacial structure more ordered (with enhanced mobility).

Supplementary Table 2. The average Bader charge transfer Δq for the Bi atoms at the stoichiometric *vs.* reduced GB. In comparison with the stoichiometric GB, the smaller average charge transfer for the Bi atoms at the reduced GB suggests weaker bonding, thereby explaining the increased kinetics of the reduced GB.

	**Stoichiometric GB**	**Reduced GB**
**Δq_{Bi} (|e|)**	1.40	0.69

Supplementary Note 10:

Enhanced grain growth in reduced atmospheres supporting the proposed mechanism

To further support proposed mechanism that the reduction can enhance grain growth, we conducted a series of controlled grain growth experiments in air, Ar, and Ar + 5% H_2, respectively by isothermally annealing samples at 880 °C for 4 hours and quenching (Supplementary Fig. 19).

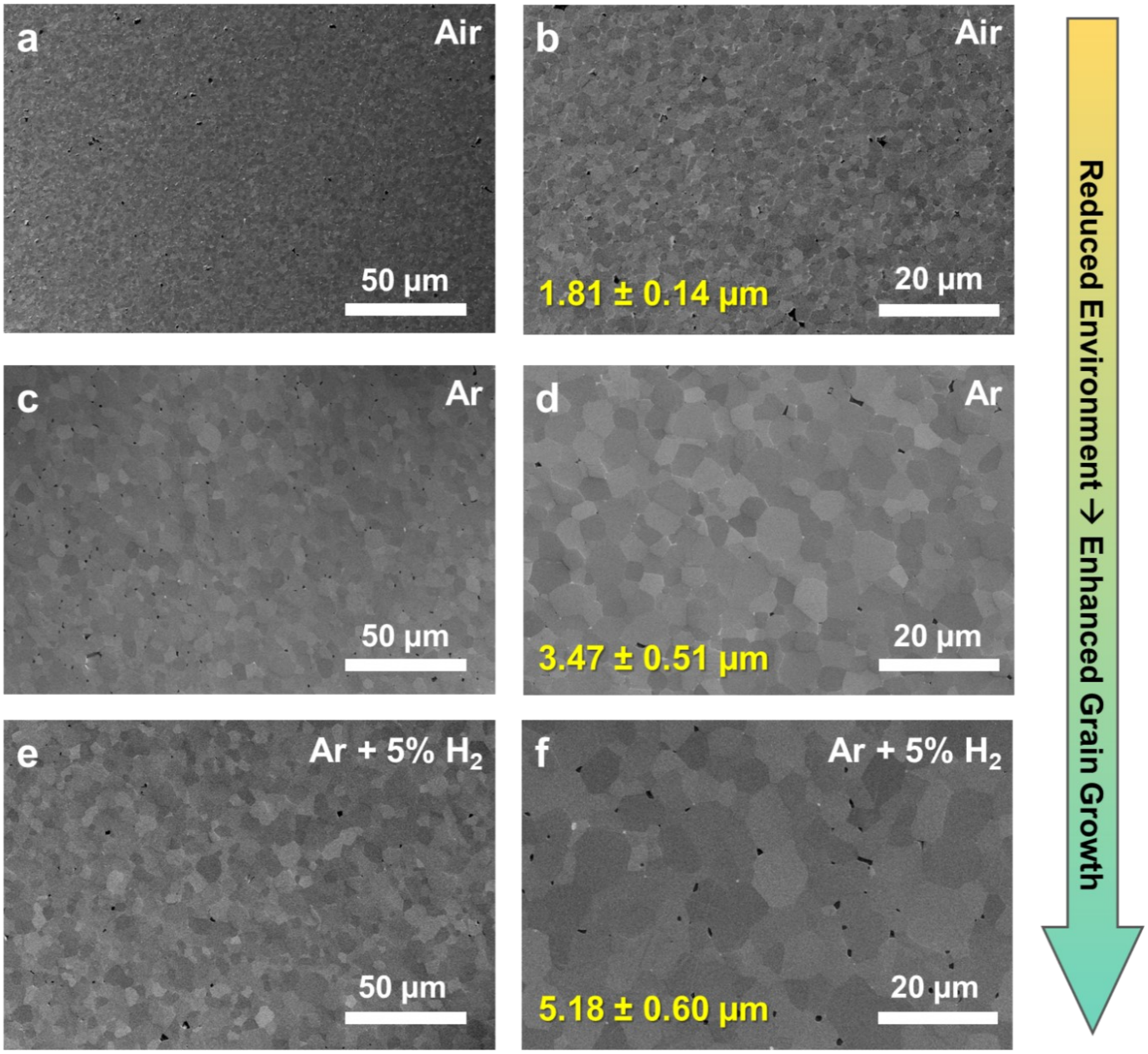

Supplementary Fig. 19. Cross-sectional SEM micrographs of three ZnO + 0.5 mol% Bi_2O_3 specimens annealed in different atmospheres, showing that a reduced environment can promote grain growth (without an applied electric current). Low- and high-magnification cross-sectional SEM micrographs of polycrystalline specimens quenched from 880 °C after annealing for 4 hours in (**a, b**) air, (**c, d**) Ar, and (**e, f**) in Ar + 5% H_2, respectively. The measured average grain sizes (± one standard deviations) are 1.81 ± 0.14 μm, 3.47 ± 0.51 μm, and 5.18 ± 0.60 μm, respectively.

The average grain size was measured to be 2.02 ± 0.17 μm for the specimen annealed in (the oxidized) air (Supplementary Fig. 19a). The measured grain size increased to 3.47 ± 0.51 μm for the specimen annealed in (the inert/reduced) Ar (Supplementary Fig. 19b), and it further increased to 5.18 ± 0.60 μm for the specimen annealed in the most reduced Ar + 5% H_2 atmosphere (Supplementary Fig. 19c).

Thus, this observation of enhanced grain growth in reduced atmospheres further supports our hypothesis that reduction can promote grain growth (even without an applied electric field/current), which are also supported by AIMD calculations of GB diffusivities (Fig. 6c).

Supplementary Note 11:

Electric characteristics of the specimens

During our isothermal annealing experiments, electric potentials and currents were recorded using a high-precision digital multimeter (Tektronix DMM 4050, Beaverton, Oregon, USA) during the experiments. The measured resistance *vs.* time curve for the PC/SC/PC sandwich specimen is shown in Supplementary Fig. 20b. For comparison, we also measured the resistivity *vs.* time curves for both a polycrystal and a single crystal (Supplementary Fig. 20a). While the resistances and resistivities changed with time initially due to evolution of defects and microstructures as well as polarization, they reached steady states after ~50 mins. At the steady state, the total resistance of the PC/SC/PC sandwich specimen was identical to that calculated from the measured resistivities of the polycrystal and the single crystal within the measurement errors.

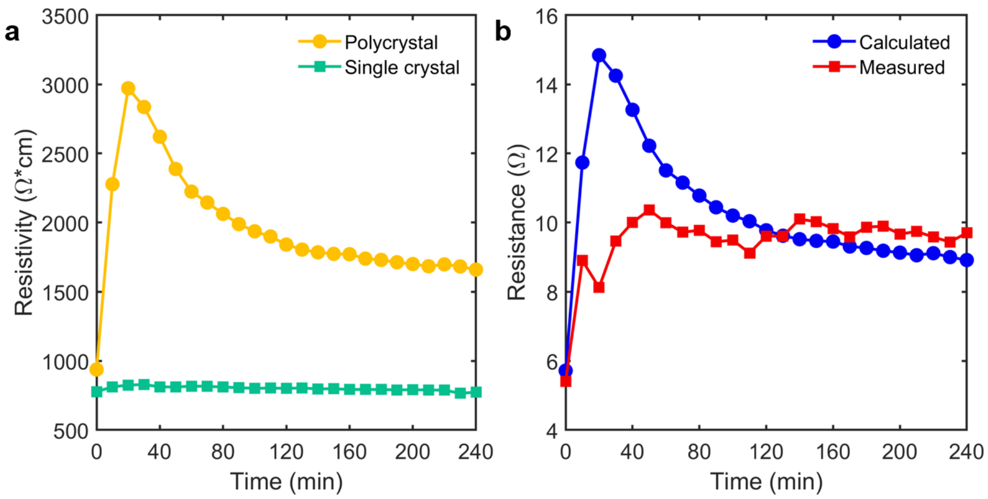

Supplementary Fig. 20. The resistance *vs.* annealing time of a sandwich specimen, a single crystal, and a polycrystal specimen isothermally annealed at a furnace temperature of 840 °C with the identical constant current density $J = 6.4$ mA/mm^2. a, The measured resistivity of the Bi_2O_3-doped ZnO polycrystal and single crystal *vs*. annealing time. **b**, The measured resistance of a PC1/SC/PC2 sandwich specimen (characterized in this study) and the calculated resistance (from the measured resistivities of the single crystal and polycrystal shown in Panel (a) and the geometry of the sandwich specimen) *vs*. annealing time. The thicknesses of PC1, SC, and PC2 of this specimen are 0.565, 0.5, and 0.565 mm, respectively. After reaching a steady state at ~50 minutes, the measured and calculated resistances of the sandwich specimens largely agree with each other. The estimated steady-state electric fields are 9.2 V/cm in the polycrystal sections (PC1 and PC2) and 9.9 V/cm for single crystal (SC) section, respectively, of the sandwich specimen.

The steady-state electric fields were calculated to be ~9.2 V/cm in the polycrystal sections (PC1 and PC2) and ~9.9 V/cm for single crystal (SC) section, respectively, of the sandwich specimen.

Supplementary References:

1 Yu, M., Grasso, S., McKinnon, R., Saunders, T. & Reece, M. J. Review of flash sintering: Materials, mechanisms and modelling. *Advances in Applied Ceramics* **116**, 24-60, doi:10.1080/17436753.2016.1251051 (2017).

2 Cologna, M., Rashkova, B. & Raj, R. Flash sintering of nanograin zirconia in <5 s at 850°c. *Journal of the American Ceramic Society* **93**, 3556-3559, doi:10.1111/j.1551-2916.2010.04089.x (2010).

3 Kim, S.-W., Kang, S.-J. L. & Chen, I. W. Electro-sintering of yttria-stabilized cubic zirconia. *Journal of the American Ceramic Society* **96**, 1398-1406, doi:10.1111/jace.12291 (2013).

4 Kim, S.-W., Kang, S.-J. L. & Chen, I. W. Ionomigration of pores and gas bubbles in yttria-stabilized cubic zirconia. *Journal of the American Ceramic Society* **96**, 1090-1098, doi:10.1111/jace.12233 (2013).

5 Munir, Z. A., Anselmi-Tamburini, U. & Ohyanagi, M. The effect of electric field and pressure on the synthesis and consolidation of materials: A review of the spark plasma sintering method. *Journal of Materials Science* **41**, 763-777, doi:10.1007/s10853-006-6555-2 (2006).

6 Castro, R. H. R. & van Benthem, K. *Sintering* **35** (2013).

7 Luo, J. The scientific questions and technological opportunities of flash sintering: From a case study of zno to other ceramics. *Scripta Materialia* **146**, 260-266, doi: 10.1016/j.scriptamat.2017.12.006 (2018).

8 Dancer, C. E. J. Flash sintering of ceramic materials. *Materials Research Express* **3**, 102001, doi:10.1088/2053-1591/3/10/102001 (2016).

9 Zhang, Y., Jung, J.-I. & Luo, J. Thermal runaway, flash sintering and asymmetrical microstructural development of zno and $ZnO–Bi_2O_3$ under direct currents. *Acta Materialia* **94**, 87-100, doi: 10.1016/j.actamat.2015.04.018 (2015).

10 Todd, R. I., Zapata-Solvas, E., Bonilla, R. S., Sneddon, T. & Wilshaw, P. R. Electrical characteristics of flash sintering: Thermal runaway of joule heating. *Journal of the European Ceramic Society* **35**, 1865-1877, doi:10.1016/j.jeurceramsoc.2014.12.022 (2015).

11 Dong, Y. & Chen, I. W. Predicting the onset of flash sintering. *Journal of the American Ceramic Society* **98**, 2333-2335, doi:10.1111/jace.13679 (2015).

12 Zhang, Y., Nie, J., Chan, J. M. & Luo, J. Probing the densification mechanisms during flash sintering of zno. *Acta Materialia* **125**, 465-475, doi: 10.1016/j.actamat.2016.12.015 (2017).

13 Ji, W. *et al.* Ultra-fast firing: Effect of heating rate on sintering of 3YSZ, with and without an electric field. *Journal of the European Ceramic Society* **37**, 2547-2551, doi:10.1016/j.jeurceramsoc.2017.01.033 (2017).

14 Kim, S.-W., Kim, S. G., Jung, J.-I., Kang, S.-J. L. & Chen, I. W. Enhanced grain boundary mobility in yttria-stabilized cubic zirconia under an electric current. *Journal of the American Ceramic Society* **94**, 4231-4238, doi:10.1111/j.1551-2916.2011.04800.x (2011).

15 Qin, W., Majidi, H., Yun, J. & van Benthem, K. Electrode effects on microstructure formation during flash sintering of yttrium-stabilized zirconia. *Journal of the American Ceramic Society* **99**, 2253-2259, doi:10.1111/jace.14234 (2016).

16 Jha, S. K., Lebrun, J. M., Seymour, K. C., Kriven, W. M. & Raj, R. Electric field induced texture in titania during experiments related to flash sintering. *Journal of the European Ceramic Society* **36**, 257-261, 10.1016/j.jeurceramsoc.2015.09.002 (2016).

17 Jha, S. K. *et al.* The effects of external fields in ceramic sintering. *Journal of the American Ceramic Society* **102**, 5-31, doi:10.1111/jace.16061 (2019).

18 Narayan, J. Grain growth model for electric field-assisted processing and flash sintering of materials. *Scripta Materialia* **68**, 785-788 (2013).

19 Chaim, R., Chevallier, G., Weibel, A. & Estournès, C. Grain growth during spark plasma and flash sintering of ceramic nanoparticles: A review. *Journal of Materials Science* **53**, 3087-3105 (2018).

20 Charalambous, H. *et al.* Inhomogeneous reduction and its relation to grain growth of titania during flash sintering. *Scripta Materialia* **155**, 37-40 (2018).

21 Biesuz, M. & Sglavo, V. M. Current-induced abnormal and oriented grain growth in corundum upon flash sintering. *Scripta Materialia* **150**, 82-86 (2018).

22 Lebrun, J.-M. *et al.* Emergence and extinction of a new phase during on–off experiments related to flash sintering of 3ysz. *Journal of the American Ceramic Society* **98**, 1493-1497, doi:10.1111/jace.13476 (2015).

23 Jha, S. K., Lebrun, J. M. & Raj, R. Phase transformation in the alumina–titania system during flash sintering experiments. *Journal of the European Ceramic Society* **36**, 733-739, doi: 10.1016/j.jeurceramsoc.2015.10.006 (2016).

24 Akdoğan, E. K. *et al.* Anomalous lattice expansion in yttria stabilized zirconia under simultaneous applied electric and thermal fields: A time-resolved in situ energy dispersive X-ray diffractometry study with an ultrahigh energy synchrotron probe. *Journal of Applied Physics* **113**, 233503, doi:10.1063/1.4811362 (2013).

25 Akdogan, O. & Dempsey, N. M. Rapid production of highly coercive Sm-Co thin films by triode sputtering. *Journal of Applied Physics* **115**, 17E508, doi:10.1063/1.4863491 (2014).

26 JomMorisaki, N., Yoshida, H., Tokunaga, T., Sasaki, K. & Yamamoto, T. Consolidation of undoped, monoclinic zirconia polycrystals by flash sintering. *Journal of the American Ceramic Society* **100**, 3851-3857, doi:10.1111/jace.14954 (2017).

27 Dong, Y. H., Wang, H. R. & Chen, I. W. Electrical and hydrogen reduction enhances kinetics in doped zirconia and ceria: I. Grain growth study. *Journal of the American Ceramic Society* **100**, 876-886, doi:10.1111/jace.14615 (2017).

28 Yoshida, H., Biswas, P., Johnson, R. & Mohan, M. K. Flash-sintering of magnesium aluminate spinel ($MgAl_2O_4$) ceramics. *Journal of the American Ceramic Society* **100**, 554-562, doi:10.1111/jace.14616 (2017).

29 Tyrpekl, V. *et al.* On the role of the electrical field in spark plasma sintering of UO_{2+x}. *Scientific Reports* **7**, 46625 (2017).

30 Zhang, J., Meng, F., Todd, R. I. & Fu, Z. The nature of grain boundaries in alumina fabricated by fast sintering. *Scripta Materialia* **62**, 658-661, doi:10.1016/j.scriptamat.2010.01.019 (2010).

31 Krause, A. R. *et al.* Review of grain boundary complexion engineering: Know your boundaries. *Journal of the American Ceramic Society* **102**, 778-800, doi:10.1111/jace.16045 (2019).

32 Zhang, Y., Nie, J. & Luo, J. Flash sintering activated by bulk phase and grain boundary complexion transformations. *Acta Materialia* **181**, 544-554, doi:10.1016/j.actamat.2019.10.009 (2019).

33 Kim, S. W., Kim, S. G., Jung, J. I., Kang, S. J. L. & Chen, I. W. Enhanced grain boundary mobility in yttria-stabilized cubic zirconia under an electric current. *Journal of the American Ceramic Society* **94**, 4231-4238, doi:10.1111/j.1551-2916.2011.04800.x (2011).

34 Grasso, S. *et al.* Flash spark plasma sintering (FSPS) of and SiC. *Journal of the American Ceramic Society* **99**, 1534-1543, doi:10.1111/jace.14158 (2016).

35 Olevsky, E. A., Rolfing, S. M. & Maximenko, A. L. Flash (ultra-rapid) spark-plasma sintering of silicon carbide. *Scientific Reports* **6**, 33408, doi:10.1038/srep33408 (2016).

36 Gild, J., Kaufmann, K., Vecchio, K. & Luo, J. Reactive flash spark plasma sintering of high-entropy ultrahigh temperature ceramics. *Scripta Materialia* **170**, 106-110, doi: 10.1016/j.scriptamat.2019.05.039 (2019).

37 Wang, C. *et al.* A general method to synthesize and sinter bulk ceramics in seconds. *Science* **368**, 521-526, doi:10.1126/science.aaz7681 (2020).

38 Dong, Y. & Chen, I. W. Oxygen potential transition in mixed conducting oxide electrolyte. *Acta Materialia* **156**, 399-410, doi: 10.1016/j.actamat.2018.06.014 (2018).

39 Sierra, J. *et al.* In-operando observation of microstructural evolution in a solid oxide cell electrolyte operating at high polarization. *Journal of Power Sources* **413**, 351-359 (2019).

40 Laguna-Bercero, M. A., Campana, R., Larrea, A., Kilner, J. A. & Orera, V. M. Electrolyte degradation in anode supported microtubular yttria stabilized zirconia-based solid oxide steam electrolysis cells at high voltages of operation. *Journal of Power Sources* **196**, 8942-8947, doi:10.1016/j.jpowsour.2011.01.015 (2011).

41 Tian, H.-K., Liu, Z., Ji, Y., Chen, L.-Q. & Qi, Y. Interfacial electronic properties dictate Li dendrite growth in solid electrolytes. *Chemistry of Materials* **31**, 7351-7359 (2019).

42 Conrad, H. & Yang, D. Dependence of the sintering rate and related grain size of yttria-stabilized polycrystalline zirconia (3Y-TZP) on the strength of an applied dc electric field. *Mater. Sci. Eng. A* **528**, 8523-8529, doi:10.1016/j.msea.2011.08.022 (2011).

43 Yang, D. & Conrad, H. Enhanced sintering rate and finer grain size in yttria-stablized zirconia (3Y-TZP) with combined dc electric field and increased heating rate. *Materials Science and Engineering a-Structural Materials Properties Microstructure and Processing* **528**, 1221-1225, doi:10.1016/j.msea.2010.10.041 (2011).

44 Obare, J., Griffin, W. D. & Conrad, H. Effects of heating rate and dc electric field during sintering on the grain size distribution in fully sintered tetragonal zirconia polycrystals stabilized with 3% molar yttria (3y-tzp). *Journal of Materials Science* **47**, 5141-5147, doi:10.1007/s10853-012-6391-5 (2012).

45 Conrad, H. Space charge and grain boundary energy in zirconia (3Y-TZP). *Journal of the American Ceramic Society* **94**, 3641-3642, doi:10.1111/j.1551-2916.2011.04823.x (2011).

46 Yang, D. & Conrad, H. Enhanced sintering rate of zirconia (3Y-TZP) by application of a small ac electric field. *Scripta Materialia* **63**, 328-331, doi:10.1016/j.scriptamat.2010.04.030 (2010).

47 Dong, Y. H., Qi, L., Li, J. & Chen, I. W. A computational study of yttria-stabilized zirconia: II. Cation diffusion. *Acta Materialia* **126**, 438-450, doi:10.1016/j.actamat.2017.01.008 (2017).

48 Dong, Y., Wang, H. & Chen, I. W. Electrical and hydrogen reduction enhances kinetics in doped zirconia and ceria: I. Grain growth study. *Journal of the American Ceramic Society* **100**, 876-886 (2017).

49 Dong, Y., Qi, L., Li, J. & Chen, I.-W. A computational study of yttria-stabilized zirconia: I. Using crystal chemistry to search for the ground state on a glassy energy landscape. *Acta Materialia* **127**, 73-84 (2017).

50 Rheinheimer, W., Fülling, M. & Hoffmann, M. J. Grain growth in weak electric fields in strontium titanate: Grain growth acceleration by defect redistribution. *Journal of the European Ceramic Society* **36**, 2773-2780, doi: j.jeurceramsoc.2016.04.033 (2016).

51 Hughes, L. A. & van Benthem, K. Effects of electrostatic field strength on grain-boundary core structures in $SrTiO_3$. *Journal of the American Ceramic Society* **102**, 4502-4510 (2019).

52 Hughes, L., Marple, M. & van Benthem, K. Electrostatic fields control grain boundary structure in $SrTiO_3$. *Applied Physics Letters* **113**, 041604 (2018).

53 Rheinheimer, W., Parras, J. P., Preusker, J.-H., De Souza, R. A. & Hoffmann, M. J. Grain growth in strontium titanate in electric fields: The impact of space-charge on the grain-boundary mobility. *Journal of the American Ceramic Society* **102**, 3779-3790, doi:10.1111/jace.16217 (2019).

54 Zhang, Y. Y. & Luo, J. Promoting the flash sintering of zno in reduced atmospheres to achieve nearly full densities at furnace temperatures of < 120 °C. *Scripta Materialia* **106**, 26-29, doi:10.1016/j.scriptamat.2015.04.027 (2015).

55 Lee, J.-R., Chiang, Y.-M. & Ceder, G. Pressure-thermodynamic study of grain boundaries: Bi segregation in ZnO. *Acta Materialia* **45**, 1247-1257 (1997).

56 Özgür, Ü. *et al.* A comprehensive review of ZnO materials and devices. *Journal of Applied Physics* **98**, 11 (2005).

57 Shuk, P., Wiemhöfer, H.-D., Guth, U., Göpel, W. & Greenblatt, M. Oxide ion conducting solid electrolytes based on Bi_2O_3. *Solid State Ionics* **89**, 179-196 (1996).

58 Per Kofstad, a. T. N. Defects and transport in crystalline solids, 2007.

59 Matsumoto, T. *et al.* Correlation between grain size and optical properties in zinc oxide thin films. *Applied Physics Letters* **81**, 1231-1233, doi:10.1063/1.1499991 (2002).

60 Fonoberov, V. A., Alim, K. A., Balandin, A. A., Xiu, F. & Liu, J. Photoluminescence investigation of the carrier recombination processes in ZnO quantum dots and nanocrystals. *Physical Review B* **73**, 165317, doi:10.1103/PhysRevB.73.165317 (2006).

61 Hofmann, D. M. *et al.* Properties of the oxygen vacancy in zno. *Applied Physics A* **88**, 147-151, doi:10.1007/s00339-007-3956-2 (2007).

62 Auret, F. D., Goodman, S. A., Legodi, M. J., Meyer, W. E. & Look, D. C. Electrical characterization of vapor-phase-grown single-crystal ZnO. *Applied Physics Letters* **80**, 1340-1342, doi:10.1063/1.1452781 (2002).

63 Van de Walle, C. G. Defect analysis and engineering in zno. *Physica B: Condensed Matter* **308-310**, 899-903, doi:10.1016/S0921-4526(01)00830-4 (2001).

64 Xiong, G., Pal, U. & Serrano, J. G. Correlations among size, defects, and photoluminescence in ZnO nanoparticles. *Journal of Applied Physics* **101**, 024317, doi:10.1063/1.2424538 (2007).

65 Gomi, M., Oohira, N., Ozaki, K. & Koyano, M. Photoluminescent and structural properties of precipitated ZnO fine particles. *Japanese Journal of Applied Physics* **42**, 481-485, doi:10.1143/jjap.42.481 (2003).

66 Kim, S., Somaratne, R. M. D. S. & Whitten, J. E. Effect of adsorption on the photoluminescence of zinc oxide nanoparticles. *The Journal of Physical Chemistry C* **122**, 18982-18994, doi:10.1021/acs.jpcc.8b04715 (2018).

67 Chiang, Y.-M., Wang, H. & Lee, J.-R. Hrem and stem of intergranular films at zinc oxide varistor grain boundaries. *Journal of Microscopy* **191**, 275-285 (1998).

68 Kaplan, W. D., Chatain, D., Wynblatt, P. & Carter, W. C. A review of wetting versus adsorption, complexions, and related phenomena: The rosetta stone of wetting *Journal of Materials Science* **48**, 5681-5717 (2013).

69 Baram, M., Chatain, D. & Kaplan, W. D. Nanometer-thick equilibrium films: The interface between thermodynmaics and atomistics. *Science* **332**, 206-209 (2011).

70 Gupta, V. K., Yoon, D.-H., Meyer, H. M. & Luo, J. Thin intergranular films and solid-state activated sintering in nickel-doped tungsten. *Acta Materialia* **55**, 3131-3142, doi:10.1016/j.actamat.2007.01.017 (2007).

71 Clarke, D. R. On the equilibrium thickness of intergranular glass phases in ceramic materials. *Journal of the American Ceramic Society* **70**, 15-22 (1987).

72 Cannon, R. M. *et al.* Adsorption and wetting mechanisms at ceramic grain boundaries. *Ceramic Transactions (Grain Boundary Engineering in Ceramics)* **118**, 427-444 (2000).

73 Luo, J., Wang, H. & Chiang, Y.-M. Origin of solid-state activated sintering in Bi_2O_3-doped ZnO. *Journal of the American Ceramic Society* **82**, 916-920, doi:10.1111/j.1151-2916.1999.tb01853.x (1999).

74 Wang, H. & Chiang, Y.-M. Thermodynamic stability of intergranular amorphous films in bismuth-doped zinc oxide. *Journal of the American Ceramic Society* **81**, 89-96 (1998).

75 Hu, T., Yang, S., Zhou, N., Zhang, Y. & Luo, J. Role of disordered bipolar complexions on the sulfur embrittlement of nickel general grain boundaries. *Nature Communications* **9**, 2764, doi:10.1038/s41467-018-05070-2 (2018).

76 Yang, S., Zhou, N., Zheng, H., Ong, S. P. & Luo, J. First-order interfacial transformations with a critical point: Breaking the symmetry at a symmetric tilt grain boundary. *Physical review letters* **120**, 085702 (2018).

77 Chua, A. L., Benedek, N. A., Chen, L., Finnis, M. W. & Sutton, A. P. A genetic algorithm for predicting the structures of interfaces in multicomponent systems. *Nature Materials* **9**, 418 (2010).

78 Hu, C., Zuo, Y., Chen, C., Ping Ong, S. & Luo, J. Genetic algorithm-guided deep learning of grain boundary diagrams: Addressing the challenge of five degrees of freedom. *Materials Today*, doi: 10.1016/j.mattod.2020.03.004 (2020).

79 Tang, M., Carter, W. C. & Cannon, R. M. Grain boundary transitions in binary alloys. *Physical Review Letters* **97**, 075502 (2006).

80 Tang, M., Carter, W. C. & Cannon, R. M. Diffuse interface model for structural transitions of grain boundaries. *Physical Review B* **73**, 024102 (2006).

81 Tang, M., Carter, W. C. & Cannon, R. M. Grain boundary order-disorder transitions. *Journal of Materials Science* **41**, 7691-7695 (2006).

82 Wang, L., Maxisch, T. & Ceder, G. Oxidation energies of transition metal oxides within the GGA + U framework. *Physical Review B* **73**, 195107, doi:10.1103/PhysRevB.73.195107 (2006).

83 Luo, J. & Chiang, Y.-M. Existence and stability of nanometer-thick disordered films on oxide surfaces. *Acta materialia* **48**, 4501-4515 (2000).
